# Using Functional Signatures to Identify Repositioned Drugs for Breast, Myelogenous Leukemia and Prostate Cancer

**DOI:** 10.1371/journal.pcbi.1002347

**Published:** 2012-02-09

**Authors:** Daichi Shigemizu, Zhenjun Hu, Jui-Hung Hung, Chia-Ling Huang, Yajie Wang, Charles DeLisi

**Affiliations:** 1 Bioinformatics Program, Boston University, Boston, Massachusetts, United States of America; 2 Laboratory of Clinical Medical Research, Department of Clinical Laboratory Diagnostics, Beijing Tiantan Hospital, Capital Medical University, Beijing, People's Republic of China; Stanford University, United States of America

## Abstract

The cost and time to develop a drug continues to be a major barrier to widespread distribution of medication. Although the genomic revolution appears to have had little impact on this problem, and might even have exacerbated it because of the flood of additional and usually ineffective leads, the emergence of high throughput resources promises the possibility of rapid, reliable and systematic identification of approved drugs for originally unintended uses. In this paper we develop and apply a method for identifying such repositioned drug candidates against breast cancer, myelogenous leukemia and prostate cancer by looking for inverse correlations between the most perturbed gene expression levels in human cancer tissue and the most perturbed expression levels induced by bioactive compounds. The method uses variable gene signatures to identify bioactive compounds that modulate a given disease. This is in contrast to previous methods that use small and fixed signatures. This strategy is based on the observation that diseases stem from failed/modified cellular functions, irrespective of the particular genes that contribute to the function, i.e., this strategy targets the functional signatures for a given cancer. This function-based strategy broadens the search space for the effective drugs with an impressive hit rate. Among the 79, 94 and 88 candidate drugs for breast cancer, myelogenous leukemia and prostate cancer, 32%, 13% and 17% respectively are either FDA-approved/in-clinical-trial drugs, or drugs with suggestive literature evidences, with an FDR of 0.01. These findings indicate that the method presented here could lead to a substantial increase in efficiency in drug discovery and development, and has potential application for the personalized medicine.

## Author Summary

The effective drug of a given disease is aimed to bring abnormal functions associated with disease back to the normal state. Using expression profile as the surrogate marker of the cellular function, we introduce a novel procedure to identify candidate therapeutics by searching for those bioactive compounds that either down-regulate abnormally over-expressed genes, or up-regulate those that are abnormally under-expressed. We show that the approach detects a pool of plausible candidates as repositioning/new drugs. In contrast to previous studies, our approach uses a variable big number of genes and/or gene combinations as a representation of functional signatures to identify bioactive compounds that modulate a given disease, irrespective of the particular genes that contribute to the cellular functions; therefore it covers potential drugs with heterogeneous properties. The method may also have potential application for the personalized medicine.

## Introduction

The average research and development (R&D) cost for the 10-odd years to develop a new pharmaceutical now exceeds a billion dollars [1], [2]; anti-cancer drugs being especially costly [2]. The process encompasses compound identification, toxicity testing in animals, early phase clinical trails, and efficacy in late phase trials. The failure of more than 90% of drugs during development [1], is perhaps the single greatest contributor to overall cost of pharmaceutical R&D. This cost in time and money can in principle be substantially reduced by repositioning drugs that are already approved for other purposes.

One way to screen approved drugs for new purposes is computationally. Computational chemistry provides valuable contributions in hit- and lead-compound discovery [3]. Systems biology approaches have also been recently used to capture the complexity of drug discovery and repositioning [4], [5]. Computational approaches have rarely, however, been a key contributor to drug discovery or repositioning [3]. This is in part because the majority of the studies focus only a few genes/proteins [6], either as the drug targets, or “disease signatures” while there is increasing evidence that many effective drugs act on multiple rather than single targets [4], and evidence is starting to emerge that pathologies can be a consequence of small abnormalities in many genes, rather than major abnormalities in a few genes [7], [8]. In addition, many existing methods constrain search space by imposing similarity requirements–including similarity of ligand structures [9], expression profile of drug response [10], topological similarity of target-drug, drug-drug and disease-drug [11], [12] networks, and side-effect similarity [13], which diminishes the effectiveness of *de novo* drug discovery.

The main idea underlying a number of current methods, including the one presented here, is to identify genes whose expressions are reverse correlated under disease and drug perturbations [14], [15], [16]. Our approach, however, uses functional signatures rather than gene signatures. Ideally a functional signature would be represented by pathways or other functional modules that are perturbed by the disease and restored by drugs. The utility of such a definition is limited by lack of a comprehensive set of functional modules/pathways. We therefore adapted an alternative approach that identifies a drug for repositioning when the reverse ordered lists of disease perturbed and drug perturbed genes has a statistically significant overlap. We thereby remove the requirement for representing a disease by a fixed number of genes. Because we use a large number of genes in our analysis, we filter out genes that are expressed differently between untreated cell line and disease samples; a step that is generally not present in gene signature based methods.

Our approach allows the detection of heterogeneous drug candidates that may restore cellular functions through different paths, in keeping with the idea that drugs acting selectively on multiple targets may be more efficacious than single-target agents, and that a particular physiological process may be modulated by multiple paths. This is in contrast to other approaches which either use a fixed small number of genes as the disease signature [14], [15], [16] or limit candidates to drugs whose properties (such as expression profiles) are similar to those of existing drugs [9], [10], [11], [12], [13].

**Figure 1:**
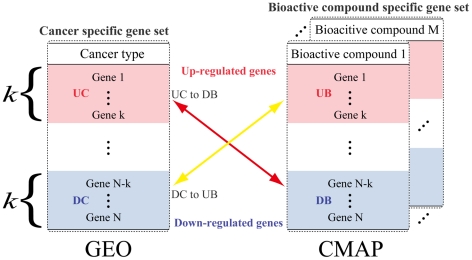
The comparison of up- or down-regulated genes between each pair of the gene-expression signature from the CMAP and the gene-expression signature from the GEO. The parameter k indicates the preselected number of up- or down-regulated genes. UC (Top ranking genes in a Cancer type) and UB (Top ranking genes with a Bioactive compound) represent up-regulated genes in the GEO and the CMAP respectively, whereas DC (Botom ranking genes in a Cancer type) and DB (Bottom ranking gene with a Bioactive compound) represent down-regulated genes in the GEO and the CMAPs respectively.

As with other approaches [14], [15], [16] we utilize two databases: the Connectivity Map (CMAP) which provides information on expressed genes in cancer cell lines perturbed by bioactive compounds [6], [14], and the Gene Expression Omnibus (GEO) [17], which stores transcript levels for various cancers. We consider as potential candidates, compounds that down (up)-regulate cell-line genes which are up (down)-regulated in transformed tissue cells. We use a three-step strategy to identify candidate compounds. First, we compare the expression of genes in the untreated cell line and the cancer tissue sample, and retain genes that are expressed in both. Second, we download the ranked list of perturbed cell line genes from CMAP, and generate a ranked list of genes from tissue samples ranked by differential expression. Both steps are designed to make the expression data comparable between cell lines and cancer samples. Finally, as shown in Fig. 1, we compare the *K* (window size) most up-regulated genes in the tissue (UC) against the *K* most down-regulated genes in the cell line list (DB), for each compound. We assume a compound is a candidate for repositioning if there is significant number of overlapping genes between UC and DB, and *vice versa*.

We illustrate that this new strategy with database integration and straightforward statistical analysis is able to identify a remarkably large number of plausible candidates for myelogenous leukemia, prostate and breast cancer. Of the more than 1300 CMAP compounds, 4 are currently in use against breast cancer, 5 against myelogenous leukemia and 3 against prostate cancer. Our analysis returned 1 of the 4, 2 of the 5 and 1 of the 3. The relative plausibility of the candidates is further indicated by the fact that 11/45, 5/50 and 6/50 candidates for repositioning against breast cancer, myelogenous leukemia and prostate cancer, respectively, are currently in clinical trials for those diseases, these statistics summarizing the most important indicators of performance. These results not only demonstrate the effectiveness of the approach, but also hint the potential application of the approach for the personalized medicine by reverse-correlating of patient's expression profile against the expression profiles of all available drugs, as detailed in the discussion section.

## Results

### Statistics of significant bioactive compounds

#### Breast cancer

As shown in Table 1, we detected 28 bioactive compounds from correlations between genes that are up-regulated in cancer (UC) and down-regulated in response to bioactive compounds (DB), and another 62 by comparing genes down-regulated in cancer (DC) to those up-regulated by bioactive compounds (UB). Of the 90, 80 either up-regulate down-regulated cancer genes (DC/UB), or down-regulate up-regulated cancer genes (UC/DB); another 10 display duality; i.e. they do both. Consequently, we identified 80 distinct compounds; 46 of them are FDA approved. CMAP includes 4 FDA approved drugs for breast cancer. We recovered one of them, fulvestrant, which displays duality (Table 2 and Table S1); i.e. it down-regulates genes that are highly up-regulated in breast cancer, and also up-regulates genes that are highly down-regulated. The remaining 45 are FDA approved for diseases other than breast cancer and are therefore candidates for repositioning.

**Table 1:** Bioactive compounds identified with optimal parameters.

Total compounds in CMAP	911	840	858
**Target Disease**	**Breast Cancer**	**Myelogenous leukemia**	**Prostate Cancer**
Compounds that are FDA drugs	509	460	482
**UC/DB**			
FDA drug in CMAP for target disease	4	5	3
Optimized parameter size	1200	700	7000
Total Predictions (a)	28	89	83
Predictions that are FDA drugs for other disease	12	47	44
Predicted FDA drugs for target disease (b)	1	2	1
Predictions with other supporting evidence (c)	9 (4 in clinical trials)	8 (5 in clinical trial)	13 (5 in clinical trials)
Total no. of predictions having supporting evidence (b+c)	10	10	14
Predictions for which trials failed (d)	2	1	1
Predicted compounds of unknown efficacy (f) [a = b+c+d+f]	16	78	68
**DC/UB**			
Optimized parameter size	1400	800	5200
Total predictions (a)	62	26	88
Predictions that are FDA drugs for other disease	38	11	50
Predicted FDA drugs for target disease (b)	1	1	1
Predictions with other supporting evidence (c)	19 (10 in clinical trials)	4	13 (6 in clinical trials)
Total no. of predictions having supporting evidence (b+c)	20	5	14
Predictions for which trials failed (d)	3	1	1
Predicted compounds of unknown efficacy (f) [a = b+c+d+f]	39	20	73

Among total 1309 compounds in CMAP, 913 (510 are FDA approved drugs) were used in this study. Supporting evidence is based on direct literature search or the ClinicalTrial.gov database. “Total compounds in CMAP” indicates the number of compounds used in the CMAP. “Compounds that are FDA drugs” counts number of FDA approved drugs in the “Total compounds in CMAP”. “Predicted FDA drugs for target disease” counts in-use drugs for the target disease in CMAP.

**Table 2:** List of repositioning candidates for three cancers.

	Results of UC/DB	Results of DC/UB
Breast Cancer	Fulvestrant*^§^, Amiloride*, Dizocilpine^§^, Estradiol* (Ph3), Irinotecan* (Ph 2)^§^, Metergoline, Nocodaole, Sirolimus* (Ph 2)^§^,Thioridazine*, Valproic acid*(Ph2)^§^, Amoxicillin*, Promethazine*, Adenosine phosphate, Benperidol^§^, Benserazide, Chlortetracycline*^§^, Desoxycortone^§^, Dexibuprofen, Domperidone*, Galantamine, Nilutamide*^§^, Pirinixic acid, Propranolol*, Rolitetracycline, Tiletamine, Troleandomycin*, Xylazine, Zaprinast	Fulvestrant*^§^, Artemisinn, Bupropion*(Ph4), Dexamethasone*(Ph3), Dizocilpine^§^, Dydrogesterone*, Etoposide*(Ph2), Gabapentin*(Ph3), Irinotecan*(Ph 2)^§^, Mestranol, Methotrexate*(Ph3), Nimesulide, Nomegestrol, Novobiocin*, Prochlorperazine*(Ph3), Sirolimus*(Ph2)^§^, Testosterone*(Ph2), Valproic acid* (Ph2)^§^, Trifluoperazine*, Troglitazone*, Amiodarone*, Fluoxetine*, Hycanthone, Acenocoumarol, Amikacin*, Azacitidine*, Benperidol^§^, Betazole*, Cetirizine*, Chlorpropamide*, Chlortetracycline*^§^, Chlorzoxazone*, Clenbuterol, Clozapine*, Debrisoquine, Desoxycortone^§^, Dinoprostone*, Dioxybenzone, Domperidone^§^, Etynodiol*, Eucatropine, Felodipine*, Gentamicin*, Guaifenesin*, Guanadrel*, Iohexol, Ketanserin, Lorglumide, Mefexamide, Metampicillin, Moroxydine, Mycophenolic acid*, Naphazolin, Nicardipine*, Nifenazone, Nilutamide*^§^, Nimodipine*, Phenoxybenzamine*, Primaquine*, Tetroquinone, Topiramate*, Tubocurarine chloride*
Acute myeloid leukemia	Etoposide*^§^, Prednisone*, Alvespimycin(Ph1), Ascorbic acid(Ph2), Disulfiram*^§^, Estradiol*^§^, Etodolac*(Ph2), Nabumetone*, Tanespimycin(Ph1), Thalidomide*(Ph2), Tranexamid acid*^§^, Acemetacin, Acenocoumarol, Alfuzosin*, Alprostadil*, Amikacin*, Astemizole, Atropine methonitrate, Atropine oxide^§^, Benzocaine, Brinzolamide*^§^, Chloroquine*, Chlorphenamine*^§^, Chlorpromazine*,Ciprofloxacin*, Clenbuterol^§^, Clorgiline, Colforsin^§^, Cotinine, Dehydrocholic acid, Desipramine, Diazoxide*, Dihydroergotamine*, Dinoprost, Diperodon^§^, Dosulepin, Doxylamine, Enoxacin*, Furosemide*, Glafenine, Glipizide*, Haloperidol*, Hycanthone, Isoconazole, Isoniazid*, Ivermectin*, Loxapine*, Mafenide^§^, Mefloquine*^§^, Mepacrine^§^, Mepenzolate bromide*, Metergoline, Methylergometrine*^§^, Metitepine, Metrizamide*, Miconazole*, Minocycline*, Minoxidil*, Molsidomine, Mometasone*, Naltrexone*, Nicardipine*, Nicergoline, Nomifensine^§^, Norfloxacin*, Orciprenaline*, Oxolinic acid^§^, Oxybuprocaine*, Oxybutynin*, Pentetrazol, Pergolide*, Perphenazine*, Phenindione*, Pindolol*, Puromycin, Pyrantel^§^, Pyridoxine*, Pyrithyldione, Quinpirole, Streptomycin*, Sulfadiazine*, Sulpiride, Tamoxifen*, Thioproperazine, Thioridazine*^§^, Ticlopidine*, Triflusal, Yohimbic acid, Zaprinast^§^	Etoposide*^§^, Estradiol*^§^, Disulfiram*^§^, Nicrosamide, Nocodazole, Tranexamic acid*^§^, Atropine oxide^§^, Brinzolamide*^§^, Bromocriptine*, Chlorphenamine*^§^, Clenbuterol^§^, Colforsin^§^, Diflunisal*, Diperodon^§^, Lanatoside c, Mefloquine*^§^, Mepacrine^§^, Methylergometrine*^§^, Neomycin*, Nomifensine^§^, Oxolinic acid^§^, Pyrantel^§^, Suloctidil, Thioridazine*^§^, Zaprinast^§^
Prostate Cancer	Diethylstilbestrol*^§^, Alprostadil*(Ph2)^§^, Chenodeoxycholic acid*^§^, Danazol*^§^, Deferoxamine*^§^, Desipramine^§^, Disulfiram*^§^, Fluvastatin*^§^, Hydrocortisone*(Ph3)^§^, Mycophenolic acid*^§^, Paclitaxel*(Ph3)^§^, Sirolimus*(Ph2)^§^, Sulindac*^§^, Tanespimycin(Ph2)^§^, Nifedipine*^§^, Adiphenine^§^, Alprenolol^§^, Alverine,Amiprilose^§^, Articaine^§^, Azapropazone^§^, Beclometasone*^§^, Benzathine benzylpenicillin^§^, Biotin^§^, Brompheniramine*^§^, Cefalotin*^§^, Chlormezanone*^§^, Chlortalidone*^§^, Clorsulon^§^,Dapsone*^§^, Debrisoquine^§^, Dihydroergotamine*^§^, Dioxybenzone^§^, Disopyramide*^§^, Dizocilpine^§^, Domperidone^§^, Ethaverine^§^, Ethionamide*^§^, Flecainide*^§^, Guanabenz*^§^, Guanadrel*^§^, Homochlorcyclizine^§^, Iohexol^§^, Isoniazid*^§^, Isoxicam^§^, Levocabastine*^§^, Lidocaine*^§^, Lynestrenol^§^, Mafenide^§^, Mefexamide^§^, Memantine*^§^,Metampicillin^§^, Metergoline^§^, Metixene*^§^, Mianserin^§^, Mometasone*^§^, Moxonidine^§^, Naftifine*, Nicergoline^§^, Niclosamide^§^, Nicotinic acid*^§^, Ondansetron*^§^, Orphenadrine*^§^, Oxantel^§^, Oxyphenbutazone*^§^, Pergolide*^§^,Perphenazine*^§^, Pimozide*^§^, Propoxycaine^§^, Pyrithyldione^§^,Ribavirin*^§^, Sisomicin^§^, Spiperone^§^, Spiramycin^§^,Spironolactone*^§^, Sulfacetamide*^§^, Tacrine*^§^, Terguride^§^,Thioproperazine^§^, Tolazamide*^§^, Tolbutamide*^§^,Triflupromazine^§^, Urapidil^§^	Diethylstilbestrol*^§^, Alprostadil*(Ph2)^§^, Chenodeoxycholic acid*^§^, Ciclosporin*(Ph3), Danazol*^§^, Deferoxamine*^§^, Desipramine^§^, Disulfiram*^§^, Hydrocortisone*(Ph3)^§^, Mycophenolic acid*^§^, Paclitaxel*(Ph3)^§^, Sirolimus*(Ph2)^§^, Sulindac*^§^, Tanespimycin(Ph 2)^§^, Nifedipine*^§^, Adiphenine^§^, Alprenolol^§^, Amiprilose^§^, Articaine^§^, Azapropazone^§^, Beclometasone*^§^, Benzathine benzylpenicillin^§^, Biotin^§^, Brompheniramine*^§^, Cefalotin*^§^, Chlormezanone*^§^, Chlortalidone*^§^, Clorsulon^§^, Dapsone*^§^, Debrisoquine^§^, Demeclocycline*, Dihydroergotamine*^§^, Dioxybenzone^§^, Disopyramide*^§^,Dizocilpine^§^, Domperidone^§^, Ethaverine^§^, Ethionamide*^§^, Flecainide*^§^, Fluvastatin*^§^, Guanabenz*^§^, Guanadrel*^§^, Homochlorcyclizine^§^, Iohexol^§^, Isoniazid*^§^, Isoxicam^§^, Levocabastine*^§^, Lidocaine*^§^, Lynestrenol^§^, Mafenide^§^, Mefexamide^§^, Memantine*^§^, Metampicillin^§^, Metergoline^§^, Metixene*^§^, Mianserin^§^, Mometasone*^§^,Moxonidine^§^, Naftifine*^§^, Netilmicin*, Nicergoline^§^, Niclosamide^§^,Nicotinic acid*^§^, Ondansetron*^§^, Orphenadrine*^§^, Oxantel^§^,Oxyphenbutazone*^§^, Pergolide*, Perphenazine*^§^, Pimozide*^§^,Propoxycaine^§^, Pyrithyldione^§^, Ribavirin*^§^, Rifampicin*, Sisomicin^§^,Spiperone^§^, Spiramycin^§^,S pironolactone*^§^, Sulfacetamide*^§^,Sulfadiazine*, Tacrine*, Terguride^§^, Thioproperazine^§^,Tolazamide*^§^,Tolbutamide*, Triamterene*, Triflupromazine^§^, Urapidil^§^

FDA approved compounds are marked with (*); Compounds showing duality with (^§^); Color of candidates match to the FDA-approved drug for the corresponding cancer, e.g. Tamoxifen is FDA-approved drug for breast cancer and is predicted as a repositioning candidate for acute myeloid leukemia. Words such as “Ph2” in the bracket of some predictions indicate that the corresponding drug is in the phase 2 clinical trial according to ClinicalTrial.gov at the time when the manuscript is prepared.

#### Myelogenous leukemia

We detected 89 (UC/DB) and 26 (DC/UB) bioactive compounds for myelogenous leukemia, 96 of which are distinct (19 show duality), and of those, 52 are FDA approved. Of the five CMAP compounds currently in use against myelogenous leukemia, we recovered 2 (etoposide and prednisone), leaving 50 candidates for repositioning.

#### Prostate cancer

We detected 83 (UC/DB) and 88 (DC/UB) bioactive compounds for prostate cancer. Of the 171, 89 are distinct and 51 of these are FDA approved. We recovered one of the 3 compounds in CMAP, which are FDA approved for prostate cancer (diethylstidbestrol), leaving 50 potential candidates for repositioning.

### Supporting evidence

#### (i) Recall

As indicated above, our method recovered 1/4, 2/5 and 1/3 of the CMAP compounds that are FDA approved for breast cancer, myelogenous leukemia and prostate cancer, respectively. We also note, as outlined below (iii), less direct, but nonetheless important supporting evidence for potential efficacy of a substantial number of identified compounds.

#### (ii) Clinical trials

Twenty-two of the predicted distinct compounds that are FDA approved, and are consequently candidates for repositioning, are in fact in clinical trials: 11 for breast cancer, 5 for leukemia and 6 for prostate cancer, representing 24% (11/45), 10% (5/50) and 12% (6/50) of the distinct candidates for those diseases.

#### (iii) Other evidence

As summarized in Tables 1 and S1, published results provide suggestive evidence for the potential efficacy of an additional 13 distinct breast cancer candidates, 5 distinct leukemia candidates and 8 distinct prostate cancer candidates. Six of the 13, three of the 5 and seven of the 8 are FDA approved drugs, and are therefore candidates for repositioning.

#### (iv) Functional plausibility

We defined perturbed pathways as over-represented in genes that are significantly up or down-regulated in diseased relative normal tissue, as explained in Methods. We expect and find that for a given disease, a number of pathways is perturbed by multiple compounds. As elaborated below, identification of common processes could provide clues about cancer biogenesis, mechanism and treatment. We look for common processes using a tandem approach: starting with pathway analysis for the most specific relations, and Gene Ontology analysis to search for higher order connections. We show that although the gene sets used for reverse-correlation may be different for different drug candidates, these genes involve many functions common to the target cancer.

#### Pathways

**Figure 2:**
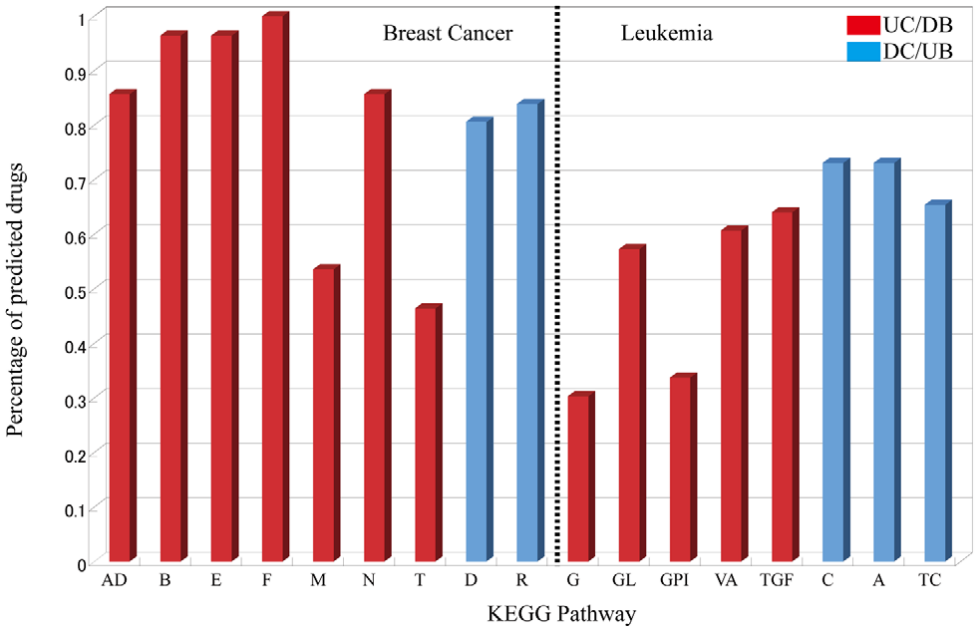
Over-represented pathways for breast cancer and myelogenous leukemia. The horizontal axis lists the pathways and the vertical axis represents the percentage of identified compounds that perturb the pathway. **AD**: Adherens junction, **B**: Bacterial invasion of epithelial cells, **E**: ErbB signaling pathway, **F**: Focal adhesion, **M**: Riboflavin metabolism, **N**: Nucleotide excision repair, **R**: Ribosome, **T**: Thiamine metabolism, **D**: Drug metabolism - cytochrome P450, **G**: Glycerolipid metabolism, **GL**: Glycerophospholipid metabolism, **GPI**: Glycosylphosphatidylinositol (GPI)-anchor biosynthesis, **VA**: Vascular smooth muscle contraction, **TGF**: TGF-βsignaling pathway, **C**: Cell cycle, **A**: Apoptosis, **TC**: T cell receptor signaling.

We mapped highly up/down regulated genes (i.e. those that are within our optimized windows (Table 1)) for a given cancer onto KEGG pathways [18], and computed the Fisher probability of chance allocation for each pathway, accepting only pathways with p-values below 0.05. We found 9 pathways over-represented in breast cancer with 7 in UC/DB and 2 in DC/UB. The same analysis for myelogenous leukemia yielded 8 pathways – 5 in UC/DB and 3 in DC/UB (Fig. 2 and Table S6). The pathways, as well as the corresponding top-ranking genes for the two cancers for identified compounds are listed in Table S2 and Table S3. For both cancers, there are no overlapping pathways between UC/DB and DC/UB, indicating clear separation of up-regulated and down-regulated pathways. The bacterial invasion of epithelial cells, ErbB signaling, and focal adhesion pathways appear to be strongly implicated in breast cancer, since almost all identified compounds strongly perturb genes that fall into each of those pathways (Fig. 2). For myelogenous leukemia, on the other hand, the most strongly implicated pathways—apoptosis and the cell cycle—each have genes that are perturbed by only slightly more than 70% of the identified compounds. There is no overlap of pathways between breast cancer and myelogenous leukemia, even though alterations appear in a number of processes common to both [19].

#### Over-represented pathways for breast cancer

Predicted drug candidates for breast cancer are aimed at restoring expression of genes that are up-regulated by the disease in seven pathways: adherens junction, focal adhesion, ErbB signaling, riboflavin metabolism, thiamine metabolism, nucleotide excision repair and bacterial invasion of epithelial cells. The inhibition of over-expressed genes in both the adherens junction and focal adhesion pathways hints at the critical role of endothelial barrier enhancement [20] to impede cancer cell extravasation. The over-representation of the bacterial invasion pathway, on the other hand, indicates augmented breast cancer cell invasiveness and adhesiveness under conditions of bacterial infection. This suggests that the increased risk of metastasis due to infection could be the result of direct interaction of infectious bacteria, and not just bacterially induced inflammation [21].

The involvement of the ErbB signaling pathway is not surprising – it is well-known that the ErbB protein family or epidermal growth factor receptor (EGFR) family, especially ErbB-2 (HER-2), is often over-expressed with aggressive clinical behavior and poor outcome in patients with breast cancer [22]. In addition, the dual inhibition of the focal adhesion and EGFR signaling pathways can cooperatively enhance apoptosis in breast cancers [23]. The identification of these pathways is consistent with the recent development of therapy for breast cancer, i.e., targeting of ErbB-2 with trastuzumab, and vascular endothelial growth factor (VEGFA) with bevacizumab in combination with chemotherapy has proven to be a milestone in molecular targeted therapy for breast cancer [24].

The over-representation of both the riboflavin (vitamin B2) metabolism and thiamine (vitamin B1) metabolism pathways is consistent with previously noted connections between vitamin B complex and breast cancer [25]. In addition the serum levels of the estrogen inducible riboflavin carrier protein, which occupies a key position in riboflavin metabolism, may be useful as a new marker to predict early-stage breast cancer [26].

Finally, the nucleotide excision repair pathway corrects DNA damage caused by environmental toxins including cigarette smoke and ultraviolet radiation. Polymorphisms in this pathway have been reported in breast cancer patients [27], suggesting the possibility of impaired repair and consequent accumulation of mutations. More specifically a number of genes in this pathway, such as ERCC4 (Table S2), are tightly associated with breast cancer [28]. In addition, one of the important cancer-related genes, P53, regulates excision repair through DNA damage response genes such as GADD45 [29].

Genes whose expression is repressed by breast cancer and increased by predicted drug candidates (DC/UB) are over-represented in only two pathways. The involvement of the cytochrome P450 (CYPs) pathway indicates that the CYPs may be key enzymes in breast cancer formation and cancer treatment. Their importance lies in the fact that they metabolize drugs used for cancer treatment, and are therefore potential targets for anticancer therapy [30]. Among our top ranking genes for predicted breast cancer drugs are CYP2A6, and CYP2C19. Their pronounced polymorphic [30] suggests that for any strategy targeting them, individualized, or stratified therapy, could be especially critical.

Disease genes that are highly perturbed are over-represented in the ribosomal pathway. Many studies report that the morphological and functional changes in the nucleolus are a consequence of both the increased demand for ribosome biogenesis, and changes in the mechanisms controlling cell proliferation. The loss or functional changes in the two major tumor suppressor proteins, retinoblastoma protein (pRB) and p53, cause an up-regulation of ribosome biogenesis in many cancer tissues including breast cancer [31]. On the other hand, some down-regulated ribosomal proteins, such as RPL35A, RPL18 and RPL14 (Table S2) that we find in both the breast cancer tissue and cell lines have received relatively little attention [32] and might be worth pursuing.

#### Over-represented pathways for myelogenous leukemia

We identified five pathways with gene sets that are highly up-regulated in myelogenous leukemia, and highly down-regulated by compounds: glycerolipid (triglyceride) metabolism, glycerophospholipid metabolism, glycosylphosphatidylinositol (GPI-anchor) biosynthesis, vascular smooth muscle contraction, and transforming growth factor β (TGF-β) signaling. The first three are components of lipid metabolism whose close association to leukemia has been studied for decades [33]. Although abnormal glycerolipid metabolism is well-known to be associated with cardiovascular disease and diabetes, there is also strong evidence that alkyl glycerolipids induce apoptosis of leukemia cell lines [34]. Disordered glycerophospholipid metabolism has been reported in the leukemia cell line and retinoic acid treatment will suppress the synthesis of ethanolamine-containing glycerophospholipids [35]. The reported connection between the synthesis of GPI-anchor and leukemia is indirect and uncommon: the deficiency of GPI-anchor occurs in rare diseases including hemolytic anemia and paroxysmal nocturnal hemoglobinuria (PNH). PNH often develops in people with aplastic anemia which occasionally transforms into leukemia [36].

The association between the TGF-β signaling pathway and cancer is well known. The pathway is involved in tumor suppression, as well as in tumor progression and invasion [37]. Its over-representation among over-expressed genes indicates that in myelogenous leukemia it more likely behaves as a promoter. This is consistent with recent observations that support a permissive role for TGF-β in growth [38] and metastasis [39] of established tumors.

There seems to be no direct association between the vascular smooth muscle (VSM) contraction pathway and leukemia/cancer; its over-representation may be the result of over-representation of genes shared by relevant pathways. In particular examination of the genes involved in the VSM pathway (Table S3) indicates that the two most frequently appearing genes: PLA2G6 and ROCK2 are also genes in GPI-anchor biosynthesis pathway and the TGF-β pathway respectively; and TGF-β is known to promote the contractile phenotype in VSM cells [40].

Three pathways have been reported among DC/UB genes; two of them, apoptosis [41] and cell cycle [42] pathways, are well known to be cancer-associated, and have been studied extensively for myelogenous leukemia. While molecular defects in apoptotic pathways are thought to often contribute to the abnormal expansion of malignant cells and their resistance to chemotherapy, the abnormality of the cell cycle pathway usually produces cells with too many or too few chromosomes (aneuploidy), which is frequently associated with the transition to leukemia. The third pathway, the T-Cell receptor signaling pathway, is central to cell-mediated immunity, which is invariably activated by tumor associated antigens [43]. The down regulated T-cell receptor signaling genes which are reactivated by the predicted drug candidates include PTPRC, CD8A, CD3D and src family protein kinase (FYN), all of which play key roles as triggering intracellular signaling including activation-induced cell death [44].

#### Gene ontology (GO) term enrichment analysis

In order to obtain broader insight we examined enriched GO terms among the identified gene sets using the GO Term Enrichment Analysis (GOTEA) and batch mode of VisANT system [45]. For the purpose of comparison, we use informative GO terms under which there are more than 400 annotated genes with FDR<0.01, and mark the terms using the abbreviation of corresponding KEGG pathways whenever they can be matched. The detailed results are listed in Table S4, S5. As expected, this analysis reveals more cellular functions, as well as the cellular compartments where these functions are carried out. Most of the over-represented pathways are reproduced. More interestingly, this analysis also finds the GO terms that are shared between UC/DB and DC/UB, probably because some of the terms, such as “regulation of transport”, are not specific enough. We also find some GO terms common to Tables S4 and Table S5, which may hint at how the drugs can be repositioned between breast cancer and myelogenous leukemia.

## Discussion

We introduced a novel procedure for identifying candidate therapeutics from gene expression profiles. The general idea is that viable drug candidates will be among those bioactive compounds that either down-regulate abnormally over-expressed genes, or up-regulate those that are abnormally under-expressed. We show that the idea leads to a pool of plausible candidates for repositioning.

### Targeting functions

One distinguishing feature of our method is that it targets cellular functions rather than genes, i.e., the focus of the method is to bring abnormal functions associated with disease back to the normal state. This strategy is based on the observation that diseases stem from failed/modified cellular functions, regardless of which of the particular genes contributing to the function are aberrant [19]. For the purpose of finding therapeutics, we do not have a fixed list of signature genes for a given disease. Instead from a large set of ranked differentially expressed genes for a particular disease, we find compounds whose effect on the expression of most perturbed genes is opposite that of the disease. This results in a number of overlapping but different (for different compounds) subsets of genes. On the other hand, for a particular disease the functions associated with the subsets are similar. This characteristic of variability at the level of genes, with conservation at the level of function can be partially seen in Table S2, S3 where for each drug candidate the list of genes is very different while the list of pathways is similar.

We used mRNA expression as a surrogate measure of the functional change because of its wide availability either for drug response or disease perturbation. The method is, however, applicable to other data types (protein expression, methylation and so fourth).

Since our method focuses on functional recovery and identifying different but overlapping subsets of genes for different compounds, it can cover potential drugs with heterogeneous properties. On the other hand, we do find genes that are targeted by a large number of our identified compounds. For example, *LAMB1*, *CAV1* and *RPL35*, tend to be targeted by most of predicted drugs for breast cancer as shown in Table S2.

### Mechanisms of action

The mechanisms and range of action of many current drugs are poorly understood. Even drugs with known targets often have “off-target” effects [5]. While many such effects are undesirable, some of them provide the opportunity for repositioning. We have used pathway analysis to interpret the functional rationale for repositioning. The same analysis also provides some understanding mechanism.

As an example, consider Tamoxifen, which is used extensively for the treatment of both early and advanced estrogen receptor positive (ER+) breast cancer [46]. Our results indicate that tamoxifen is a candidate for repositioning to myelogenous leukemia. In particular, the overrepresentation of genes in this pathway, which are upregulated in myelogenous leukemia, and down-regulated by Tamoxifen suggests the possibility that aberrant TGF-β signaling plays a role in myelogenous leukemia. Since TGF-β production is down-regulated by tamoxifen in other tissues [47], tamoxifen might function as an anti-myelogenous leukemia drug by repressing this pathway (Table S3).

This suggestion is supported by the fact that expression of estrogen receptors ESR1 and ESR2 is relatively unaffected by treatment with Tamoxifen (of the 20,469 ranked genes, ESR1 and ESR2 ranked 4184 and 4734 respectively – well below the number of top ranking genes used in the study: 700/800 for UC/DB and DC/UB). Consequently it seems unlikely that the effect of Tamoxifen on leukemic cells is mediated by these receptors.

We therefore speculate that tamoxifen acts similarly in breast cancer, and thereby exerts its effects in a dual manner; i.e. through inhibition of TGF-β, in addition to inhibition of estrogen. Militating against this possibility are the facts that the TGF-β pathway is not over-represented in UC/DB transcripts, and other investigations did not find evidence for the regulation of TGF-β genes/proteins by tamoxifen in breast cancer patients [48]. On the other hand an increased expression of TGF-β1, which is often seen in tumors of breast cancer patients, correlates with poor prognostic outcome [49]. This apparent conflict might be resolved by the recent discovery that tamoxifen decreases extracellular TGF-β1 proteins secreted from breast cancer cells, but not intracellular ones [50]. This result is also compatible with our finding that the adherens junction and focal adhesion pathways are both over-represented in breast cancer cells, and these pathways are potentially inducible by TGF-β [37]. These observation are in line with other studies documenting decreased metastasis when TGF-β signalling is blocked in high-grade breast tumor [51], and suggest that tamoxifen represses the metastasis of breast cancer cells by down regulating the TGF-β pathway and preventing loss of polarity and cell–cell contacts.

Taken collectively, the functional analysis of our results suggests a potential mechanism for tamoxifen, which is independent of an interaction with the estrogen receptor, and has tamoxifen suppressing tumor metastasis and growth by down-regulating TGF-β signaling.

### Beyond repositioning

Our results also suggest that some exploration of the identified non-FDA approve drugs (new drug candidates) could be fruitful. If the fraction of FDA approved drugs in clinical trials is taken as a measure of what is worth exploring (i.e. we conservatively neglect other supporting evidence), then we'd expect 8 of the 34 non-FDA approved drugs for breast cancer to be ultimately worthy of clinical trials; and 4 of the 44 for myelogenous leukemia and 5 of the 38 for prostate cancer (i.e. we'd expect this number to get through animal toxicity tests, and efficacy tests when available, and enter phase 1 trials).

### Limitation and future development

There are several issues that may limit the future development of the approach. First, the optimization of the window size requires availability of the known FDA-approved drugs in CMAP, which may not always the case especially when expanding this approach to the other diseases that are functionally close to the three cancers. Second, the sensitivity of the approach to the subtype, or the different stage, of the same disease needs to be studied further. The approach will have great application to the personalized medicine if it is able to identify different drugs for the disease at different stages/subtype because the relative cheap price to get the patient expression profile. Finally, although mRNA expression is used to measure the functional change of the cell, we expected the better results using the other data that may be more representative of the cellular functions, such as protein expressions.

## Materials and Methods

### Transcript expression

Expression data in response to bioactive compounds for breast cancer, prostate cancer and myelogenous leukemia cell lines were obtained from the connectivity map (http://www.broad.mit.edu/CMAP/) (Build 02) [6], [14]. Differential expression data in response to breast cancer (GDS2617), leukemia (GDS2908), and prostate cancer (GDS1439) were obtained from the National Center for Biotechnology Information (NCBI) Gene Expression Omnibus (GEO) [17]. The data sets are picked in such a way that there is fairly big number of samples and the expressions are normalized by GEO database. The ranked list of differentially expressed genes for a given cancer is calculated using t-statistic.

### Gene filtering

The bioactive compound specific signatures fetched from CMAP are based on cell lines (i.e. cancerous cells with and without treatments), while those from GEO were based on tissue cells (i.e. normal and cancer tissue cells). Since the different cell types are not directly comparable, we first normalized gene-expressions according to the untreated cell line and the cancer tissue samples. We retain only genes that are expressed in both tissue and cell line. In particular we applied the t-test to the normalized scores, and calculated the corrected p-values for multiple testing by a false discovery rate (FDR) procedure. The FDR is defined as the expected proportion of false positives among the significant results and is a more appropriate measure than the raw p-value for multiple hypotheses testing. The FDR threshold was set as 0.01, and the genes with clearly different gene-expressions were removed from both samples. As a result, we retained 15572 genes (77%), 20469 genes (92%), and 12220 genes (55%) for breast cancer, myelogenous leukemia, and prostate cancer, respectively.

### Comparison of reverse-correlated cancer and bioactive compound specific gene sets

We prepared two types of ranked lists of genes. One was generated from tissue samples ranked by differential expression between normal and cancer tissues from GEO data. The other was obtained from the ranked list of perturbed cell line genes from CMAP. In the former case, the top and bottom k genes were defined as up-regulated genes in cancer (UC) and down-regulated genes in cancer (DC). In the latter case, the top and bottom k genes were defined as up-regulated genes by bioactive compounds (UB) and down-regulated genes by bioactive compounds (DB). The genes of interest are the top and the bottom k genes in a ranked list where k ranges from 100 to 10000 in increments of 100.

We counted overlapping genes in between UC and DB (UC/DB) and in between DC and UB (DC/UB) to investigate compounds up-regulating down-regulated cancer genes (DC/UB), or down-regulating up-regulated cancer genes (UC/DB). We performed the Fisher's exact test to prove if the overlap is significant by comparing the number of overlapping genes to that of randomly selecting genes (background). The p-value was transformed into FDA corrected for multiple hypotheses. The FDR threshold was set as 0.01.

### Choice of window size

For each value k, a compound is labeled as bioactive if the number of overlapping genes (as explained in Fig. 1) is statistically significant. The sensitivity and specificity were calculated by measuring the proportions of true positives (fraction of FDA drugs identified) and true negatives (fraction of identified compounds that failed clinical trials). For each cancer, we chose values of k (one for UC/DB and one for DC/UB) that gave maximum specificity, subject to the constraint of non zero sensitivity (at least 1 correct prediction), non zero duality and a FDR less than 0.01. In this way we identified for further investigation, a total of 90 compounds (and associated genes) for breast cancer (28 suppressors of up-regulated cancer genes; 62 enhancers of down-regulated genes); 36 compounds for myelogenous leukemia (10 suppressors; 26 enhancers), and 171 compounds for prostate cancer (83 suppressors; 88 activators). The results regarding different window size are presented in Table S7 and Fig. S1.

### Pathway over-representation analysis

We mapped correlated genes in UC/DB and in DC/UB onto the KEGG pathways and counted the number of genes mapped and total number of existing genes with respect to each pathway. Given the number of genes and total number of all of genes we used, a p-value is calculated with hypergeometic distribution [52]; we accepted only pathways with the p-values below 0.05 as over-represented pathways [53].

### Drug and clinical trail information retrieval

We collected data from KEGG DRUG Database (http://www.genome.jp/kegg/drug/), DrugBank (http://www.drugbank.ca/) and PharmGKB (http://www.pharmgkb.org/) to map International Nonproprietary Name (INN) to generic names and alias. FDA approved drugs were found from FDA service: Drugs@FDA. All clinical trials data and references that we checked for our predictions were shown in Table 2 and Table S1 with corresponding hyperlinks.

## Supporting Information

Figure S1 The specificity and the sensitivity against bioactive compounds identified in each parameter *k* with respect to each cancer type for both with and without filtering out genes with apparently different gene-expressions in between different cell types. (A) Breast cancer with filtering (B) Breast cancer without filtering (C) Leukemia with filtering (D) Leukemia without filtering (E) Prostate cancer with filtering (F) Prostate cancer without filtering.
(JPG)

Table S1 Candidates for repositioning for three cancers. FDA approved compounds (*); Compounds showing duality (^§^); The 1^st^ number in the bracket associated with each compound is the p-value, the 2^nd^ number is the number of overlapping genes.
(DOC)

Table S2 KEGG pathways enriched in top up/down regulated genes breast cancer tissue and corresponding down/up regulated genes in response to cell line perturbations with bioactive compounds (see Methods). **AD**: Adherens junction, **B**: Bacterial invasion of epithelial cells, **D**: Drug metabolism - cytochrome P450, **E**: ErbB signaling pathway, **F**: Focal adhesion, **M**: Riboflavin metabolism, **N**: Nucleotide excision repair, **R**: Ribosome, **T**: Thiamine metabolism.
(DOC)

Table S3 KEGG pathways enriched in top up/down regulated genes leukemia and corresponding down/up regulated genes in response to cell line perturbations with bioactive compounds (see Methods). **G**: Glycerolipid metabolism, **GL**: Glycerophospholipid metabolism, **GPI**: Glycosylphosphatidylinositol (GPI)-anchor biosynthesis, **VA**: Vascular smooth muscle contraction, **TGF**: TGF-*β* signaling pathway, **C**: Cell cycle, **A**: Apoptosis, **TC**: T cell receptor signaling.
(DOC)

Table S4 GO terms enriched in top up/down regulated genes in breast cancer tissue for the window size specified in Table 1.
(DOC)

Table S5 GO terms enriched in top up/down regulated genes in leukemic tissue for the window size specified in Table 1.
(DOC)

Table S6 Enriched KEGG pathways for breast cancer and leukemia and the corresponding p-value.
(DOC)

Table S7 Sensitivity and the specificity for optimal values of window size, k.
(DOC)

## References

[1] Chong CR, Sullivan DJ (2007). New uses for old drugs.. Nature.

[2] Kamb A, Wee S, Lengauer C (2007). Why is cancer drug discovery so difficult?. Nat Rev Drug Discov.

[3] Schneider G (2010). Virtual screening: an endless staircase?. Nat Rev Drug Discov.

[4] Hopkins AL (2008). Network pharmacology: the next paradigm in drug discovery.. Nat Chem Biol.

[5] Berger SI, Iyengar R (2009). Network analyses in systems pharmacology.. Bioinformatics.

[6] Lamb J (2007). The Connectivity Map: a new tool for biomedical research.. Nat Rev Cancer.

[7] Schadt EE, Friend SH, Shaywitz DA (2009). A network view of disease and compound screening.. Nat Rev Drug Discov.

[8] Yang TH, Kon M, Hung JH, Delisi C (2011). Combinations of newly confirmed Glioma-Associated loci link regions on chromosomes 1 and 9 to increased disease risk.. BMC Med Genomics.

[9] Keiser MJ, Setola V, Irwin JJ, Laggner C, Abbas AI (2009). Predicting new molecular targets for known drugs.. Nature.

[10] Iorio F, Bosotti R, Scacheri E, Belcastro V, Mithbaokar P (2010). Discovery of drug mode of action and drug repositioning from transcriptional responses.. Proc Natl Acad Sci U S A.

[11] Chiang AP, Butte AJ (2009). Systematic evaluation of drug-disease relationships to identify leads for novel drug uses.. Clin Pharmacol Ther.

[12] Hu G, Agarwal P (2009). Human disease-drug network based on genomic expression profiles.. PLoS One.

[13] Campillos M, Kuhn M, Gavin AC, Jensen LJ, Bork P (2008). Drug target identification using side-effect similarity.. Science.

[14] Lamb J, Crawford ED, Peck D, Modell JW, Blat IC (2006). The Connectivity Map: using gene-expression signatures to connect small molecules, genes, and disease.. Science.

[15] Dudley JT, Sirota M, Shenoy M, Pai RK, Roedder S (2011). Computational repositioning of the anticonvulsant topiramate for inflammatory bowel disease.. Sci Transl Med.

[16] Sirota M, Dudley JT, Kim J, Chiang AP, Morgan AA (2011). Discovery and preclinical validation of drug indications using compendia of public gene expression data.. Sci Transl Med.

[17] Barrett T, Suzek TO, Troup DB, Wilhite SE, Ngau WC (2005). NCBI GEO: mining millions of expression profiles–database and tools.. Nucleic Acids Res.

[18] Kanehisa M, Araki M, Goto S, Hattori M, Hirakawa M (2008). KEGG for linking genomes to life and the environment.. Nucleic Acids Res.

[19] Linghu B, Snitkin ES, Hu Z, Xia Y, Delisi C (2009). Genome-wide prioritization of disease genes and identification of disease-disease associations from an integrated human functional linkage network.. Genome Biol.

[20] Sun X, Shikata Y, Wang L, Ohmori K, Watanabe N (2009). Enhanced interaction between focal adhesion and adherens junction proteins: involvement in sphingosine 1-phosphate-induced endothelial barrier enhancement.. Microvasc Res.

[21] Lu H, Ouyang W, Huang C (2006). Inflammation, a key event in cancer development.. Mol Cancer Res.

[22] Franklin MC, Carey KD, Vajdos FF, Leahy DJ, de Vos AM (2004). Insights into ErbB signaling from the structure of the ErbB2-pertuzumab complex.. Cancer Cell.

[23] Golubovskaya VM, Zheng M, Zhang L, Li JL, Cance WG (2009). The direct effect of focal adhesion kinase (FAK), dominant-negative FAK, FAK-CD and FAK siRNA on gene expression and human MCF-7 breast cancer cell tumorigenesis.. BMC Cancer.

[24] Alvarez RH (2010). Present and future evolution of advanced breast cancer therapy.. Breast Cancer Res.

[25] Liu S, Huang H, Lu X, Golinski M, Comesse S (2003). Down-regulation of thiamine transporter THTR2 gene expression in breast cancer and its association with resistance to apoptosis.. Mol Cancer Res.

[26] Rao PN, Levine E, Myers MO, Prakash V, Watson J (1999). Elevation of serum riboflavin carrier protein in breast cancer.. Cancer Epidemiol Biomarkers Prev.

[27] Rajaraman P, Bhatti P, Doody MM, Simon SL, Weinstock RM (2008). Nucleotide excision repair polymorphisms may modify ionizing radiation-related breast cancer risk in US radiologic technologists.. Int J Cancer.

[28] Milne RL, Ribas G, Gonzalez-Neira A, Fagerholm R, Salas A (2006). ERCC4 associated with breast cancer risk: a two-stage case-control study using high-throughput genotyping.. Cancer Res.

[29] Smith ML, Chen IT, Zhan Q, Bae I, Chen CY (1994). Interaction of the p53-regulated protein Gadd45 with proliferating cell nuclear antigen.. Science.

[30] Ingelman-Sundberg M, Sim SC, Gomez A, Rodriguez-Antona C (2007). Influence of cytochrome P450 polymorphisms on drug therapies: pharmacogenetic, pharmacoepigenetic and clinical aspects.. Pharmacol Ther.

[31] Belin S, Beghin A, Solano-Gonzalez E, Bezin L, Brunet-Manquat S (2009). Dysregulation of ribosome biogenesis and translational capacity is associated with tumor progression of human breast cancer cells.. PLoS One.

[32] Lukk M, Kapushesky M, Nikkila J, Parkinson H, Goncalves A (2010). A global map of human gene expression.. Nat Biotechnol.

[33] Pallasch CP, Schwamb J, Konigs S, Schulz A, Debey S (2008). Targeting lipid metabolism by the lipoprotein lipase inhibitor orlistat results in apoptosis of B-cell chronic lymphocytic leukemia cells.. Leukemia.

[34] Vogler WR, Olson AC, Hajdu J, Shoji M, Raynor R (1993). Structure-function relationships of alkyl-lysophospholipid analogs in selective antitumor activity.. Lipids.

[35] Naito M, Kudo I, Nakagawa Y, Waku K, Nojiri H (1991). Suppression of ethanolamine-containing glycerophospholipid synthesis in HL-60 cells during retinoic acid-induced differentiation.. J Biochem.

[36] Parker C, Omine M, Richards S, Nishimura J, Bessler M (2005). Diagnosis and management of paroxysmal nocturnal hemoglobinuria.. Blood.

[37] Ikushima H, Miyazono K (2010). TGFbeta signalling: a complex web in cancer progression.. Nat Rev Cancer.

[38] Naka K, Hoshii T, Muraguchi T, Tadokoro Y, Ooshio T (2010). TGF-beta-FOXO signalling maintains leukaemia-initiating cells in chronic myeloid leukaemia.. Nature.

[39] Horiguchi K, Shirakihara T, Nakano A, Imamura T, Miyazono K (2009). Role of Ras signaling in the induction of snail by transforming growth factor-beta.. J Biol Chem.

[40] Risinger GM, Updike DL, Bullen EC, Tomasek JJ, Howard EW (2010). TGF-beta suppresses the upregulation of MMP-2 by vascular smooth muscle cells in response to PDGF-BB.. Am J Physiol Cell Physiol.

[41] Stahnke K, Eckhoff S, Mohr A, Meyer LH, Debatin KM (2003). Apoptosis induction in peripheral leukemia cells by remission induction treatment in vivo: selective depletion and apoptosis in a CD34+ subpopulation of leukemia cells.. Leukemia.

[42] Fimognari C, Lenzi M, Sciuscio D, Cantelli-Forti G, Hrelia P (2007). Cell-cycle specificity of sulforaphane-mediated apoptosis in Jurkat T-leukemia cells.. In Vivo.

[43] Heemskerk MH (2010). T-cell receptor gene transfer for the treatment of leukemia and other tumors.. Haematologica.

[44] Unsinger J, Herndon JM, Davis CG, Muenzer JT, Hotchkiss RS (2006). The role of TCR engagement and activation-induced cell death in sepsis-induced T cell apoptosis.. J Immunol.

[45] Hu Z, Hung JH, Wang Y, Chang YC, Huang CL (2009). VisANT 3.5: multi-scale network visualization, analysis and inference based on the gene ontology.. Nucleic Acids Res.

[46] (EBCTCG). EBCTCG (2005). Effects of chemotherapy and hormonal therapy for early breast cancer on recurrence and 15-year survival: an overview of the randomised trials.. Lancet.

[47] Kuhn MA, Wang X, Payne WG, Ko F, Robson MC (2002). Tamoxifen decreases fibroblast function and downregulates TGF(beta2) in dupuytren's affected palmar fascia.. J Surg Res.

[48] Brandt S, Kopp A, Grage B, Knabbe C (2003). Effects of tamoxifen on transcriptional level of transforming growth factor beta (TGF-beta) isoforms 1 and 2 in tumor tissue during primary treatment of patients with breast cancer.. Anticancer Res.

[49] Ivanovic V, Demajo M, Krtolica K, Krajnovic M, Konstantinovic M (2006). Elevated plasma TGF-beta1 levels correlate with decreased survival of metastatic breast cancer patients.. Clin Chim Acta.

[50] Nilsson UW, Jonsson JA, Dabrosin C (2009). Tamoxifen decreases extracellular TGF-beta1 secreted from breast cancer cells–a post-translational regulation involving matrix metalloproteinase activity.. Exp Cell Res.

[51] Pardali K, Moustakas A (2007). Actions of TGF-beta as tumor suppressor and pro-metastatic factor in human cancer.. Biochim Biophys Acta.

[52] Beissbarth T, Speed TP (2004). GOstat: find statistically overrepresented Gene Ontologies within a group of genes.. Bioinformatics.

[53] Curtis RK, Oresic M, Vidal-Puig A (2005). Pathways to the analysis of microarray data.. Trends Biotechnol.

