# Supplementary figures and images for: Using Functional Signatures to Identify Repositioned Drugs for Breast, Myelogenous Leukemia and Prostate Cancer
Source: PLoS Comput Biol. 2012 Feb 9;8(2):e1002347. doi: 10.1371/journal.pcbi.1002347 (PMC3276504; doi:10.1371/journal.pcbi.1002347)

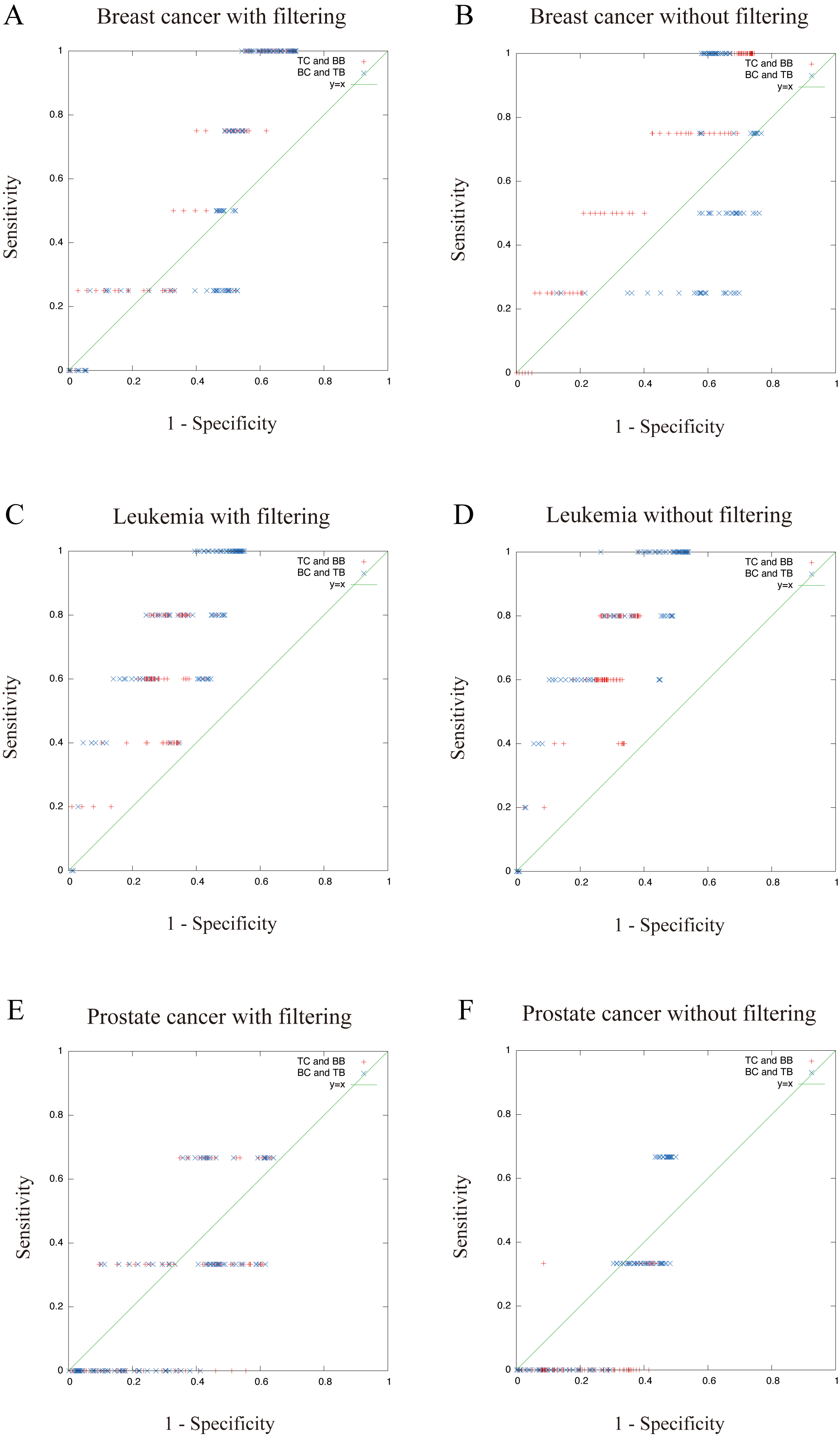

Supplement: Figure S1 — The specificity and the sensitivity against bioactive compounds identified in each parameter k with respect to each cancer type for both with and without filtering out genes with apparently different gene-expressions in between different cell types. (A) Breast cancer with filtering (B) Breast cancer without filtering (C) Leukemia with filtering (D) Leukemia without filtering (E) Prostate cancer with filtering (F) Prostate cancer without filtering. (JPG) [file pcbi.1002347.s001.jpg]
