# Supplementary material for: Using Functional Signatures to Identify Repositioned Drugs for Breast, Myelogenous Leukemia and Prostate Cancer
Source: PLoS Comput Biol. 2012 Feb 9;8(2):e1002347. doi: 10.1371/journal.pcbi.1002347 (PMC3276504; doi:10.1371/journal.pcbi.1002347)
Supplement: Table S1 — Candidates for repositioning for three cancers. FDA approved compounds (*); Compounds showing duality (§); The 1st number in the bracket associated with each compound is the p-value, the 2nd number is the number of overlapping genes. (DOC) [file pcbi.1002347.s002.doc]

**Table S1.** Candidates for repositioning are shown with identical colors under different diseases. FDA approved compounds (*); Compounds showing duality (^§^); The 1^st^ number in the bracket associated with each compound is the p-value, the 2^nd^ number is the number of overlapping genes.

| Total **1309** compounds in CMAP, **913** (**510** are FDA approved drugs) used in this study | | | |
| --- | --- | --- | --- |
|  | Breast Cancer | Leukemia | Prostate Cancer |
| Results of UC/DB | | | |
| FDA drug in CMAP for target disease | Fulvestrant  Tamoxifen  Exemestane  Letrozole | Tretinoin  Pepstatin  Etoposide  Prednisone  Prednisolone | Diethylstilbestrol  Megestrol  Nilutamide |
| Predictions that are FDA drugs for other disease | Amiloride*(1e-3, 134)  Amoxicillin*(2e-3, 131)  Chlortetracycline*^§^(3e-3, 130)  Estradiol*(1e-3, 133)  Irinotecan*^§^(3e-4, 138)  Nilutamide*^§^(1e-5, 145)  Promethazine*(3e-3, 130)  Propranolol*(3e-3, 130)  Sirolimus*^§^(9e-3, 127)  Thioridazine*(1e-3, 132)  Troleandomycin*(6e-3, 128)  Valproic acid*^§^(4e-10, 163) | Alfuzosin*(2e-3, 43)  Alprostadil*(1e-3, 44)  Amikacin*(3e-9, 61)  Brinzolamide*^§^(3e-7, 56)  Chloroquine*(7e-3, 41)  Chlorphenamine*^§^(6e-9, 60)  Chlorpromazine*(4e-3, 42)  Ciprofloxacin*(4e-3, 42)  Diazoxide*(7e-4, 45)  Dihydroergotamine*(2e-3, 43)  Disulfiram*^§^(1e-5, 51)  Enoxacin*(2e-4, 47)  Estradiol*^§^(4e-11, 65)  Etodolac*(3e-4, 46)  Furosemide*(2e-5, 50)  Glipizide*(2e-4, 47)  Haloperidol*(2e-3, 43)  Isoniazid*(3e-6, 53 )  Ivermectin*(5e-5, 49)  Loxapine*(3e-6, 53)  Mefloquine*^§^(3e-7, 56)  Mepenzolate bromide*(7e-3, 41)  Methylergometrine*^§^ (7e-3, 41)  Metrizamide* (1e-3, 44)  Miconazole* (1e-4, 48)  Minocycline* (1e-4, 48)  Minoxidil* (4e-3, 42)  Mometasone* (1e-6, 54)  Nabumetone* (7e-4, 45)  Naltrexone* (3e-4, 46)  Nicardipine* (6e-7, 55)  Norfloxacin* (3e-6, 53)  Orciprenaline* (1e-3, 44)  Oxybuprocaine* (6e-6, 52)  Oxybutynin* (7e-3, 41)  Pergolide* (1e-3, 44)  Perphenazine* (3e-4, 46)  Phenindione* (7e-4, 45)  Pindolol* (4e-3, 42)  Pyridoxine* (1e-3, 44)  Streptomycin* (1e-6, 54)  Sulfadiazine* (1e-4, 48)  Tamoxifen* (3e-9, 61)  Thalidomide* (2e-4, 47)  Thioridazine*^§^ (7e-3, 41)  Ticlopidine* (5e-5, 49)  Tranexamic acid*^§^ (3e-7, 56) | Alprostadil*^§^(5e-3, 4104)  Beclometasone*^§^(4e-3, 4106)  Brompheniramine*^§^(1e-4, 4141)  Cefalotin*^§^(5e-3, 4104)  Chenodeoxycholic acid*^§^ (2e-3, 4113)  Chlormezanone*^§^(6e-3, 4102)  Chlortalidone*^§^(2e-4, 4132)  Danazol*^§^(1e-4, 4138)  Dapsone*^§^(2e-3, 4111)  Deferoxamine*^§^(1e-3, 4116)  Dihydroergotamine*^§^(7e-3, 4101)  Disopyramide*^§^(7e-3, 4100)  Disulfiram*^§^(6e-3, 4102)  Ethionamide*^§^(2e-3, 4113)  Flecainide*^§^(2e-3)  Fluvastatin*^§^(2e-4, 4134)  Guanabenz*^§^(1e-3, 4117)  Guanadrel*(9e-3, 4097)  Hydrocortisone*^§^(6e-5, 4146)  Isoniazid*^§^(2e-3, 4111)  Levocabastine*^§^(3e-3, 4109)  Lidocaine*^§^ (1e-4, 4137)  Memantine*^§^ (4e-3, 4105)  Metixene*^§^ (8e-3, 4099)  Mometasone*^§^ (4e-3, 4105)  Mycophenolic acid*^§^ (1e-3, 4119)  Naftifine*^§^ (7e-4, 4124)  Nicotinic acid*^§^ (2e-4, 4132)  Nifedipine*^§^ (2e-3, 4114)  Ondansetron*^§^ (4e-3, 4107)  Orphenadrine*^§^ (5e-3, 4104)  Oxyphenbutazone*^§^ (5e-3, 4104)  Paclitaxel*^§^ (1e-4, 4142)  Pergolide*^§^ (8e-3, 4099)  Perphenazine*^§^ (8e-4, 4123)  Pimozide*^§^ (3e-3, 4108)  Ribavirin*^§^ (4e-3, 4107)  Sirolimus*^§^ (1e-4, 4142)  Spironolactone*^§^ (7e-3, 4101)  Sulfacetamide*^§^ (4e-3, 4107)  Sulindac*^§^ (6e-4, 4126)  Tacrine*^§^ (7e-3, 4100)  Tolazamide*^§^ (4e-3, 4107)  Tolbutamide*^§^ (2e-6, 4170) |
| Predicted FDA drugs for target disease | Fulvestrant*^§^ (1e-3, 133) | Etoposide*^§^ (7e-3, 41)  Prednisone* (5e-12, 67) | Diethylstilbestrol*^§^ (7e-3, 4101) |
| Predictions with other supporting evidence | [Amiloride*](http://www.ncbi.nlm.nih.gov/pubmed/10824746) (1e-3, 134)  [Dizocilpine](http://www.springerlink.com/content/4316j55q05086706/)^§^ (6e-4, 136)  [Estradiol* (Ph 3)](http://clinicaltrials.gov/ct2/show/NCT00193726?term=ESTRADIOL&cond=breast+cancer&rank=15) (1e-3, 133)  [Irinotecan* (Ph 2)](http://clinicaltrials.gov/ct2/show/NCT00003351?term=IRINOTECAN&cond=breast+cancer&rank=6)^§^ (3e-4, 138)  [Metergoline](http://www.springerlink.com/content/w76t077740163737/) (1e-3, 133)  [Nocodaole](http://www.jci.org/articles/view/13275/version/1) (3e-3, 130)  [Sirolimus* (Ph 2)](http://clinicaltrials.gov/ct2/show/NCT00411788?term=SIROLIMUS&cond=breast+cancer&rank=1)^§^ (9e-3, 127)  [Thioridazine*](http://jpet.aspetjournals.org/content/263/1/186.abstract) (1e-3, 132)  [Valproic acid*(Ph 2)](http://clinicaltrials.gov/ct2/show/NCT01010854?term=VALPROIC+ACID&cond=breast+cancer&rank=2)^§^ (4e-10, 163) | [Alvespimycin(Ph 1)](http://clinicaltrials.gov/ct2/show/NCT01126502?term=ALVESPIMYCIN&cond=leukemia&phase=0123&rank=1) (2e-4, 47)  [Ascorbic acid(Ph 2)](http://clinicaltrials.gov/ct2/show/NCT00184054?term=ASCORBIC&cond=leukemia&rank=1) (3e-9, 61)  [Disulfiram*](http://www.ncbi.nlm.nih.gov/pubmed/17026967)^§^ (1e-5, 51)  [Estradiol*](http://www.jbc.org/content/277/47/44772.abstract)^§^ (4e-11, 65)  [Etodolac*(Ph 2)](http://clinicaltrials.gov/ct2/show/NCT00151736?term=ETODOLAC&cond=leukemia&phase=0123&rank=1) (3e-4, 46)  [Nabumetone*](http://www.ncbi.nlm.nih.gov/pubmed/16019514) (7e-4, 45)  [Tanespimycin(Ph 1)](http://clinicaltrials.gov/ct2/show/NCT00100997?term=TANESPIMYCIN&cond=leukemia&phase=0123&rank=3) (7e-4, 45)  [Thalidomide*(Ph 2)](http://clinicaltrials.gov/ct2/show/NCT00006226?term=THALIDOMIDE&cond=leukemia&phase=0123&rank=2) (2e-4, 47) | [Alprostadil*(Ph2)](http://clinicaltrials.gov/ct2/show/NCT00080808?term=ALPROSTADIL&cond=prostate+cancer&rank=2) ^§^ (5e-3, 4104)  [Chenodeoxycholic acid*](http://www.ncbi.nlm.nih.gov/pubmed/12969788)^§^  (2e-3, 4113)  [Danazol*](http://www3.interscience.wiley.com/journal/112723117/abstract)^§^ (1e-4, 4138)  [Deferoxamine*](http://www.informaworld.com/smpp/content~db=all~content=a790502556)^§^ (1e-3, 4116)  [Desipramine](http://www.ncbi.nlm.nih.gov/pubmed/18606486)^§^ (4e-5, 4152)  [Disulfiram*](http://webcache.googleusercontent.com/search?q=cache:NF7tcE11YBcJ:www.asco.org/ASCOv2/Meetings/Abstracts%3F%26vmview%3Dabst_detail_view%26confID%3D64%26abstractID%3D20452+"DISULFIRAM+"+"prostate+cancer"&cd=1&hl=ja&ct=clnk&gl=jp&client=firefox-a)^§^ (6e-3, 4102 )  [Fluvastatin*](http://mct.aacrjournals.org/content/5/9/2310.long)^§^ (2e-4, 4134)  [Hydrocortisone*(Ph 3)](http://clinicaltrials.gov/ct2/show/NCT00002881?term=HYDROCORTISONE&cond=prostate+cancer&rank=3)^§^ (6e-5, 4146)  [Mycophenolic acid*](http://www.ncbi.nlm.nih.gov/pubmed/16356827)^§^ (1e-3, 4119)  [Paclitaxel*(Ph 3)](http://clinicaltrials.gov/ct2/show/NCT00004054?term=PACLITAXEL&cond=prostate+cancer&rank=19)^§^ (1e-4, 4142)  [Sirolimus*(Ph 2)](http://clinicaltrials.gov/ct2/show/NCT00922129?term=SIROLIMUS&cond=prostate+cancer&rank=1)^§^ (1e-4, 4142)  [Sulindac*](http://www.ncbi.nlm.nih.gov/pubmed/9933075)^§^ (6e-4, 4126)  [Tanespimycin(Ph 2)](http://clinicaltrials.gov/ct2/show/NCT00118092?term=TANESPIMYCIN&cond=prostate+cancer&rank=2)^§^ (5e-3, 4104) |
| Predictions for which trials failed | [Amoxicillin*](http://www.patientsville.com/medication/amoxicillin_side_effects.htm) (2e-3, 131)  [Promethazine*](http://www.ncbi.nlm.nih.gov/pubmed/8746124) (3e-3, 130) | [Tranexamid acid*](http://www3.interscience.wiley.com/journal/122355552/abstract)^§^ (3e-7, 56) | [Nifedipine*](http://www.ncbi.nlm.nih.gov/pubmed/15728367)^§^ (2e-3, 4114) |
| New predictions | Adenosine phosphate (4e-3, 129)  Benperidol^§^ (3e-6, 148)  Benserazide (4e-4, 137)  Chlortetracycline*^§^ (3e-3, 130)  Desoxycortone^§^ (1e-4, 140)  Dexibuprofen (6e-4, 136)  Domperidone* (1e-3, 134)  Galantamine (2e-3, 131)  Nilutamide*^§^ (1e-5, 145)  Pirinixic acid (6e-3, 128)  Propranolol* (3e-3, 130)  Rolitetracycline (9e-3, 127)  Tiletamine (1e-3, 133)  Troleandomycin* (6e-3, 128)  Xylazine (6e-3, 128)  Zaprinast (1e-3, 134) | Acemetacin (1e-3, 44)  Acenocoumarol (1e-5, 51)  Alfuzosin* (2e-3, 43)  Alprostadil* (1e-3, 44)  Amikacin* (3e-9, 61)  Astemizole (3e-6, 53)  Atropine methonitrate (1e-4, 48)  Atropine oxide^§^ (2e-12, 68)  Benzocaine (7e-3, 41)  Brinzolamide*^§^ (3e-7, 56)  Chloroquine* (7e-3, 41)  Chlorphenamine*^§^ (6e-9, 60)  Chlorpromazine* (4e-3, 42)  Ciprofloxacin* (4e-3, 42)  Clenbuterol^§^ (2e-5, 50)  Clorgiline (1e-12, 69)  Colforsin^§^ (2e-3, 43)  Cotinine (2e-5, 50)  Dehydrocholic acid (7e-3, 41)  Desipramine (1e-3, 44)  Diazoxide* (7e-4, 45)  Dihydroergotamine* (2e-3, 43)  Dinoprost (4e-3, 42)  Diperodon^§^ (2e-4, 47)  Dosulepin (2e-4, 47)  Doxylamine (2e-3, 43)  Enoxacin* (2e-4, 47)  Furosemide* (2e-5, 50)  Glafenine (2e-3, 43)  Glipizide* (2e-4, 47)  Haloperidol* (2e-3, 43)  Hycanthone (5e-5, 49)  Isoconazole (3e-6, 53)  Isoniazid* (3e-6, 53)  Ivermectin* (5e-5, 49)  Loxapine* (3e-6, 53)  Mafenide^§^ (2e-16, 71)  Mefloquine*^§^ (3e-7, 56)  Mepacrine^§^ (2e-5, 50)  Mepenzolate bromide* (7e-3, 41)  Metergoline (7e-3, 41)  Methylergometrine*^§^ (7e-3, 41)  Metitepine (2e-4, 47)  Metrizamide* (1e-3, 44)  Miconazole* (1e-4, 48)  Minocycline* (1e-4, 48)  Minoxidil* (4e-3, 42)  Molsidomine (1e-3, 44)  Mometasone* (1e-6, 54)  Naltrexone* (3e-4, 46)  Nicardipine* (6e-7, 55)  Nicergoline (7e-3, 41)  Nomifensine^§^ (4e-3, 42)  Norfloxacin* (3e-6, 53)  Orciprenaline* (1e-3, 44)  Oxolinic acid^§^ (2e-4, 47)  Oxybuprocaine* (6e-6, 52)  Oxybutynin* (7e-3, 41)  Pentetrazol (2e-4, 47)  Pergolide* (1e-3, 44)  Perphenazine* (3e-4, 46)  Phenindione* (7e-4, 45)  Pindolol* (4e-3, 42)  Puromycin (7e-4, 45)  Pyrantel^§^ (6e-6, 52)  Pyridoxine* (1e-3, 44)  Pyrithyldione (4e-3, 42)  Quinpirole (3e-4, 46)  Streptomycin* (1e-6, 54)  Sulfadiazine* (1e-4, 48)  Sulpiride (2e-3, 43)  Tamoxifen* (3e-9, 61)  Thioproperazine (7e-4, 45)  Thioridazine*^§^ (7e-3, 41)  Ticlopidine* (5e-5, 49)  Triflusal (5e-5, 49)  Yohimbic acid (1e-3, 44)  Zaprinast^§^ (1e-5, 51) | Adiphenine^§^ (2e-4, 4133)  Alprenolol^§^ (4e-3, 4105)  Alverine (9e-3, 4097)  Amiprilose^§^ (8e-3, 4099)  Articaine^§^ (2e-3, 4113)  Azapropazone^§^ (4e-3, 4105)  Beclometasone*^§^ (4e-3)  Benzathine benzylpenicillin^§^ (2e-3, 4111)  Biotin^§^ (1e-3, 4120)  Brompheniramine*^§^ (1e-4, 4141)  Cefalotin*^§^ (5e-3, 4104)  Chlormezanone*^§^ (6e-3, 4102)  Chlortalidone*^§^ (2e-4, 4132)  Clorsulon^§^ (1e-3, 4116)  Dapsone*^§^ (2e-3, 4111)  Debrisoquine^§^ (3e-3, 4110)  Dihydroergotamine*^§^ (7e-3, 4101)  Dioxybenzone^§^ (8e-3, 4098)  Disopyramide*^§^ (7e-3, 4101)  Dizocilpine^§^ (8e-3, 4098)  Domperidone^§^ (6e-5, 4149)  Ethaverine^§^ (8e-3, 4098)  Ethionamide*^§^ (2e-3, 4113)  Flecainide*^§^ (2e-3, 4114)  Guanabenz*^§^ (1e-3, 4117)  Guanadrel*^§^ (9e-3, 4097)  Homochlorcyclizine^§^ (2e-3, 4115)  Iohexol^§^ (1e-3, 4117)  Isoniazid*^§^ (2e-3, 4111)  Isoxicam^§^ (3e-3, 4109)  Levocabastine*^§^ (3e-3, 4109)  Lidocaine*^§^ (1e-4, 4137)  Lynestrenol^§^ (1e-4, 4137)  Mafenide^§^ (3e-3, 4109)  Mefexamide^§^ (2e-3, 4115)  Memantine*^§^ (4e-3, 4105)  Metampicillin^§^ (9e-3, 4097)  Metergoline^§^ (1e-4, 4137)  Metixene*^§^ (8e-3, 4099)  Mianserin^§^ (8e-3, 4099)  Mometasone*^§^ (4e-3, 4105)  Moxonidine^§^ (1e-4, 4138)  Naftifine* (7e-4, 4124)  Nicergoline^§^ (1e-3, 4118)  Niclosamide^§^ (8e-4, 4123)  Nicotinic acid*^§^ (2e-4, 4132)  Ondansetron*^§^ (4e-3, 4107)  Orphenadrine*^§^ (5e-3, 4104)  Oxantel^§^ (2e-3, 4114)  Oxyphenbutazone*^§^ (5e-3, 4104)  Pergolide*^§^ (8e-3, 4099)  Perphenazine*^§^ (8e-4, 4123)  Pimozide*^§^ (3e-3, 4108)  Propoxycaine^§^ (8e-3, 4099)  Pyrithyldione^§^ (5e-4, 4127)  Ribavirin*^§^ (4e-3, 4107)  Sisomicin^§^ (1e-3, 4117)  Spiperone^§^ (3e-3)  Spiramycin^§^ (6e-4, 4125)  Spironolactone*^§^ (7e-3, 4101)  Sulfacetamide*^§^ (4e-3, 4107)  Tacrine*^§^ (7e-3, 4100)  Terguride^§^ (6e-5, 4147)  Thioproperazine^§^ (9e-3, 4097)  Tolazamide*^§^ (4e-3, 4107)  Tolbutamide*^§^ (2e-6, 4170)  Triflupromazine^§^ (2e-3, 4111)  Urapidil^§^ (7e-3, 4100) |
| Results of DC/UB | | | |
| Predictions that are FDA drugs for other disease | Amikacin* (1e-4, 179)  Amiodarone* (6e-4, 174)  Azacitidine* (9e-3, 163)  Betazole* (3e-3, 167)  Bupropion* (7e-3, 164)  Cetirizine* (7e-5, 181)  Chlorpropamide* (2e-3, 169)  Chlortetracycline*^§^ (9e-3, 163)  Chlorzoxazone* (2e-3, 169)  Clozapine* (7e-5, 164)  Dexamethasone* (9e-3, 163)  Dinoprostone* (6e-3, 165)  Dydrogesterone* (8e-4, 173)  Etoposide* (1e-3, 171)  Etynodiol* (8e-4, 173)  Felodipine* (7e-3, 164)  Fluoxetine* (9e-3, 163)  Gabapentin* (6e-3, 165)  Gentamicin* (6e-3, 165)  Guaifenesin* (7e-3, 164)  Guanadrel* (6e-3, 165)  Irinotecan*^§^ (2e-6, 191)  Methotrexate* (9e-3, 163)  Mycophenolic acid* (1e-5, 185)  Nicardipine* (2e-3, 169)  Nilutamide*^§^ (1e-3, 171)  Nimodipine* (7e-3, 164)  Novobiocin* (2e-3, 168)  Phenoxybenzamine* (1e-4, 179)  Primaquine* (6e-3, 165)  Prochlorperazine* (1e-3, 171)  Sirolimus*^§^ (1e-4, 179)  Testosterone* (4e-4, 175)  Topiramate* (7e-3, 164)  Trifluoperazine* (1e-3, 171)  Troglitazone* (6e-3, 165)  Tubocurarine chloride* (1e-5, 186)  Valproic acid*^§^ (5e-5, 182) | Brinzolamide* (1e-3, 56)  Bromocriptine*^§^ (3e-3, 54)  Chlorphenamine*^§^ (4e-4, 58)  Diflunisal* (6e-3, 53)  Disulfiram*^§^ (9e-3, 52)  Estradiol*^§^ (4e-4, 59)  Mefloquine*^§^ (7e-5, 62)  Methylergometrine*^§^ (9e-3, 52)  Neomycin* (3e-3, 54)  Thioridazine*^§^ (2e-4, 60)  Tranexamic acid*^§^ (4e-4, 58) | Alprostadil*^§^ (5e-3, 2306)  Beclometasone*^§^ (4e-3, 2310)  Brompheniramine*^§^ (1e-4, 2342)  Cefalotin*^§^ (4e-3, 2310)  Chenodeoxycholic acid*^§^ (3e-3, 2311)  Chlormezanone*^§^ (4e-3, 2310)  Chlortalidone*^§^ (1e-4, 2339)  Ciclosporin* (8e-3, 2301)  Danazol* (1e-4, 2338)  Dapsone* (1e-3, 2318)  Deferoxamine*^§^ (1e-3, 2320)  Demeclocycline* (9e-3, 2299)  Dihydroergotamine*^§^ (8e-3, 2301)  Disopyramide*^§^ (5e-3, 2306)  Disulfiram*^§^ (5e-3, 2306)  Ethionamide*^§^ (1e-3, 2320)  Flecainide*^§^ (2e-3, 2315)  Fluvastatin*^§^ (1e-4, 2338)  Guanabenz*^§^ (1e-3, 2318)  Guanadrel* (8e-3, 2301)  Hydrocortisone*^§^ (5e-5, 2350)  Isoniazid*^§^ (3e-3, 2312)  Levocabastine*^§^ (4e-3, 2309)  Lidocaine*^§^ (9e-5, 2345)  Memantine*^§^ (3e-3, 2312)  Metixene*^§^ (7e-3, 2302)  Mometasone*^§^ (8e-3, 2301)  Mycophenolic acid*^§^ (6e-4, 2328)  Naftifine*^§^ (7e-4, 2327)  Netilmicin* (9e-3, 2300)  Nicotinic acid*^§^ (3e-4, 2334)  Nifedipine*^§^ (2e-3, 2316)  Ondansetron*^§^ (4e-3, 2308)  Orphenadrine*^§^ (7e-3, 2302)  Oxyphenbutazone*^§^ (4e-3, 2310)  Paclitaxel*^§^ (6e-5, 2348)  Pergolide*^§^ (6e-3, 2305)  Perphenazine*^§^ (7e-4, 2327)  Pimozide*^§^ (4e-3, 2310)  Ribavirin*^§^ (4e-3, 2310)  Rifampicin* (8e-3, 2301)  Sirolimus*^§^ (1e-4, 2343)  Spironolactone* (6e-3, 2305)  Sulfacetamide*^§^ (4e-3, 2309)  Sulfadiazine* (8e-3, 2301)  Sulindac*^§^ (5e-4, 2330)  Tacrine*^§^ (5e-3, 2306)  Tolazamide*^§^ (3e-3, 2312)  Tolbutamide*^§^ (1e-6, 2376)  Triamterene* (9e-3, 2300) |
| Predicted FDA drugs for target disease | Fulvestrant*^§^ (1e-3, 170) | Etoposide*^§^ (1e-3, 56) | Diethylstilbestrol*^§^ (6e-3, 2304) |
| Predictions with other supporting evidence | [Artemisinn](http://webcache.googleusercontent.com/search?q=cache:S-7aJ2ZjuF4J:www.cbcrp.org/research/PageGrant.asp%3Fgrant_id%3D4768+"ARTEMISININ+"+"breast+cancer"&cd=4&hl=ja&ct=clnk&gl=jp&client=firefox-a) (1e-3, 171)  [Bupropion*(Ph 4)](http://clinicaltrials.gov/ct2/show/NCT00234195?term=BUPROPION&cond=breast+cancer&rank=1) (7e-3, 164)  [Dexamethasone*(Ph 3)](http://clinicaltrials.gov/ct2/show/NCT00475085?term=DEXAMETHASONE&cond=breast+cancer&rank=2) (9e-3, 163)  [Dizocilpine](http://www.springerlink.com/content/4316j55q05086706/)^§^ (1e-3, 171)  [Dydrogesterone*](http://ar.iiarjournals.org/content/24/3A/1433.abstract) (8e-4, 173)  [Etoposide*(Ph 2)](http://clinicaltrials.gov/ct2/show/NCT00810017?term=ETOPOSIDE&cond=breast+cancer&rank=1) (1e-4, 171)  [Gabapentin*(Ph 3)](http://clinicaltrials.gov/ct2/show/NCT00087399?term=GABAPENTIN&cond=breast+cancer&rank=4) (6e-3, 165)  [Irinotecan*(Ph 2)](http://clinicaltrials.gov/ct2/show/NCT00003351?term=IRINOTECAN&cond=breast+cancer&rank=6)^§^ (2e-6, 191)  [Mestranol](http://www.sciencedirect.com/science?_ob=ArticleURL&_udi=B6TC9-4FW1F9K-H&_user=489277&_coverDate=12%2F31%2F1970&_rdoc=1&_fmt=high&_orig=search&_sort=d&_docanchor=&view=c&_searchStrId=1324171716&_rerunOrigin=google&_acct=C000022679&_version=1&_urlVersion=0&_userid=489277&md5=b812fc350d7aa488ec1a1571b2a0008f) (3e-3, 167)  [Methotrexate*(Ph 3)](http://clinicaltrials.gov/ct2/show/NCT00296010?term=METHOTREXATE&cond=breast+cancer&rank=9) (9e-3, 163)  [Nimesulide](http://www.ncbi.nlm.nih.gov/pubmed/19046800) (6e-4, 174)  [Nomegestrol](http://www.ncbi.nlm.nih.gov/pubmed/15767920) (7e-3, 164)  [Novobiocin*](http://www.ncbi.nlm.nih.gov/pubmed/18261372) (2e-3, 168)  [Prochlorperazine*(Ph 3)](http://clinicaltrials.gov/ct2/show/NCT00475085?term=PROCHLORPERAZINE&cond=breast+cancer&rank=1) (1e-3, 170)  [Sirolimus*(Ph 2)](http://clinicaltrials.gov/ct2/show/NCT00411788?term=SIROLIMUS&cond=breast+cancer&rank=1)^§^ (1e-4, 179)  [Testosterone*(Ph 2)](http://clinicaltrials.gov/ct2/show/NCT00497458?term=TESTOSTERONE&cond=breast+cancer&rank=1) (4e-4, 175)  [Valproic acid* (Ph 2)](http://clinicaltrials.gov/ct2/show/NCT01010854?term=VALPROIC+ACID&cond=breast+cancer&rank=2)^§^ (5e-5, 182)  [Trifluoperazine*](http://www.ncbi.nlm.nih.gov/pubmed/8349440) (1e-3, 172)  [Troglitazone*](http://www.ncbi.nlm.nih.gov/pubmed/19699029) (6e-3, 165) | [Estradiol*](http://www.jbc.org/content/277/47/44772.abstract)^§^ (4e-4, 59)  [Disulfiram*](http://www.ncbi.nlm.nih.gov/pubmed/17026967)^§^ (9e-3, 52)  [Nicrosamide](http://cancerres.aacrjournals.org/cgi/content/full/70/6/2516) (6e-3, 53)  [Nocodazole](http://ar.iiarjournals.org/content/25/5/3321.abstract) (4e-4, 59) | [Alprostadil*(Ph 2)](http://clinicaltrials.gov/ct2/show/NCT00080808?term=ALPROSTADIL&cond=prostate+cancer&rank=2)^§^ (5e-3, 2306)  [Chenodeoxycholic acid*](http://www.ncbi.nlm.nih.gov/pubmed/12969788)^§^ (3e-3, 2311)  [Ciclosporin*(Ph 3)](http://clinicaltrials.gov/ct2/show/NCT00922129?term=CICLOSPORIN&cond=Prostate+cancer&rank=1) (8e-3, 2301)  [Danazol*](http://www3.interscience.wiley.com/journal/112723117/abstract)^§^ (1e-4, 2338)  [Deferoxamine*](http://www.informaworld.com/smpp/content~db=all~content=a790502556)^§^ (1e-3, 2320)  [Desipramine](http://www.ncbi.nlm.nih.gov/pubmed/18606486)^§^ (4e-5, 2355)  [Disulfiram*](http://webcache.googleusercontent.com/search?q=cache:NF7tcE11YBcJ:www.asco.org/ASCOv2/Meetings/Abstracts%3F%26vmview%3Dabst_detail_view%26confID%3D64%26abstractID%3D20452+"DISULFIRAM+"+"prostate+cancer"&cd=1&hl=ja&ct=clnk&gl=jp&client=firefox-a)^§^ (5e-3, 2306)  [Hydrocortisone*(Ph 3)](http://clinicaltrials.gov/ct2/show/NCT00002881?term=HYDROCORTISONE&cond=prostate+cancer&rank=3)^§^ (5e-5, 2350)  [Mycophenolic acid*](http://www.ncbi.nlm.nih.gov/pubmed/16356827)^§^ (6e-4, 2328)  [Paclitaxel*(Ph 3)](http://clinicaltrials.gov/ct2/show/NCT00004054?term=PACLITAXEL&cond=prostate+cancer&rank=19)^§^ (6e-5, 2348)  [Sirolimus*(Ph 2)](http://clinicaltrials.gov/ct2/show/NCT00922129?term=SIROLIMUS&cond=prostate+cancer&rank=1)^§^ (1e-4, 2343)  [Sulindac*](http://www.ncbi.nlm.nih.gov/pubmed/9933075)^§^ (5e-4, 2330)  [Tanespimycin(Ph 2)](http://clinicaltrials.gov/ct2/show/NCT00118092?term=TANESPIMYCIN&cond=prostate+cancer&rank=2)^§^ (5e-3, 2306) |
| Predictions for which trials failed | [Amiodarone*](http://www.ncbi.nlm.nih.gov/pubmed/7882454) (6e-4, 174)  [Fluoxetine*](http://aje.oxfordjournals.org/cgi/content/full/162/9/835) (9e-3, 163)  [Hycanthone](http://www.ncbi.nlm.nih.gov/pubmed/7448830) (9e-3, 163) | [Tranexamic acid*](http://www3.interscience.wiley.com/journal/122355552/abstract)^§^ (4e-4, 58) | [Nifedipine*](http://www.ncbi.nlm.nih.gov/pubmed/15728367)^§^ (2e-3, 2316) |
| New predictions | Acenocoumarol (3e-3, 167)  Amikacin* (1e-4, 179)  Azacitidine* (9e-3, 163)  Benperidol^§^ (2e-3, 168)  Betazole* (3e-3, 167)  Cetirizine* (7e-5, 181)  Chlorpropamide* (2e-3, 169)  Chlortetracycline*^§^ (9e-3, 163)  Chlorzoxazone* (2e-3, 168)  Clenbuterol (2e-3, 168)  Clozapine* (7e-5, 181)  Debrisoquine (7e-3, 164)  Desoxycortone^§^ (1e-4, 179)  Dinoprostone* (6e-3, 165)  Dioxybenzone (9e-3, 163)  Domperidone^§^ (2e-3, 168)  Etynodiol* (8e-4, 173)  Eucatropine (7e-3, 164)  Felodipine* (7e-3, 164)  Gentamicin* (6e-3, 165)  Guaifenesin* (7e-3, 164)  Guanadrel* (6e-3, 165)  Iohexol (9e-3, 163)  Ketanserin (1e-3, 171)  Lorglumide (4e-4, 175)  Mefexamide (2e-3, 168)  Metampicillin (3e-3, 167)  Moroxydine (7e-3, 164)  Mycophenolic acid* (1e-5, 185)  Naphazoline (2e-3, 168)  Nicardipine* (2e-3, 168)  Nifenazone (8e-4, 173)  Nilutamide*^§^ (1e-3, 171)  Nimodipine* (7e-3, 164)  Phenoxybenzamine* (1e-4, 179)  Primaquine* (6e-3, 165)  Tetroquinone (2e-3, 168)  Topiramate* (7e-3, 164)  Tubocurarine chloride* (1e-5, 186) | Atropine oxide^§^ (6e-7, 68)  Brinzolamide*^§^ (1e-3, 56)  Bromocriptine* (3e-3, 54)  Chlorphenamine*^§^ (4e-4, 58)  Clenbuterol^§^ (3e-3, 54)  Colforsin^§^ (9e-3, 52)  Diflunisal* (6e-3, 53)  Diperodon^§^ (9e-3, 52)  Lanatoside c (4e-4, 58)  Mafenide^§^ (4e-4, 59)  Mefloquine*^§^ (7e-5, 62)  Mepacrine^§^ (5e-7, 69)  Methylergometrine*^§^ (9e-3, 52)  Neomycin* (3e-3, 54)  Nomifensine^§^ (2e-3, 55)  Oxolinic acid^§^ (2e-3, 55)  Pyrantel^§^ (4e-4, 59)  Suloctidil (6e-3, 53)  Thioridazine*^§^ (2e-4, 60)  Zaprinast^§^ (4e-4, 58) | Adiphenine^§^ (3e-4, 2334)  Alprenolol^§^ (7e-3, 2303)  Amiprilose^§^ (6e-3, 2305)  Articaine^§^ (2e-3, 2314)  Azapropazone^§^ (4e-3, 2309)  Beclometasone*^§^ (4e-3, 2310)  Benzathine benzylpenicillin^§^ (2e-3, 2317)  Biotin^§^ (7e-4, 2327)  Brompheniramine*^§^ (1e-4, 2342)  Cefalotin*^§^ (4 e-3, 2310)  Chlormezanone*^§^ (4e-3, 2310)  Chlortalidone*^§^ (1e-4, 2339)  Clorsulon^§^ (1e-3, 2322)  Dapsone*^§^ (1e-3, 2318)  Debrisoquine^§^ (2e-3, 2316)  Demeclocycline* (9e-3, 2299)  Dihydroergotamine*^§^ (8e-3, 2301)  Dioxybenzone^§^ (6e-3, 2304)  Disopyramide*^§^ (5e-3, 2306)  Dizocilpine^§^ (7e-3, 2303)  Domperidone^§^ (4e-5, 2352)  Ethaverine^§^ (9e-3, 2300)  Ethionamide*^§^ (1e-3, 2320)  Flecainide*^§^ (2e-3, 2315)  Fluvastatin*^§^ (1e-4, 2338)  Guanabenz*^§^ (1e-3, 2318)  Guanadrel*^§^ (8e-3, 2301)  Homochlorcyclizine^§^ (2e-3, 2317)  Iohexol^§^ (1e-3, 2320)  Isoniazid*^§^ (3e-3, 2312)  Isoxicam^§^ (4e-3, 2310)  Levocabastine*^§^ (4e-3, 2310)  Lidocaine*^§^ (9e-5, 2345)  Lynestrenol^§^ (1e-4, 2341)  Mafenide^§^ (4e-3, 2310)  Mefexamide^§^ (1e-3, 2319)  Memantine*^§^ (3e-3, 2312)  Metampicillin^§^ (6e-3, 2304)  Metergoline^§^ (1e-4, 2341)  Metixene*^§^ (7e-3, 2302)  Mianserin^§^ (9e-3, 2300)  Mometasone*^§^ (8e-3, 2301)  Moxonidine^§^ (1e-4, 2343)  Naftifine*^§^ (7e-4, 2327)  Netilmicin* (9e-3, 2300)  Nicergoline^§^ (1e-3, 2319)  Niclosamide^§^ (5e-4, 2329)  Nicotinic acid*^§^ (3e-4, 2334)  Ondansetron*^§^ (4e-3, 2308)  Orphenadrine*^§^ (7e-3, 2302)  Oxantel^§^ (2e-3, 2316)  Oxyphenbutazone*^§^ (4e-3, 2308)  Pergolide* (6e-3, 2305)  Perphenazine*^§^ (7e-4, 2327)  Pimozide*^§^ (4e-3, 2308)  Propoxycaine^§^ (6e-3, 2305)  Pyrithyldione^§^ (4e-4, 2331)  Ribavirin*^§^ (4e-3, 2308)  Rifampicin* (8e-3, 2301)  Sisomicin^§^ (1e-3, 2320)  Spiperone^§^ (3e-3, 2313)  Spiramycin^§^ (8e-4, 2325)  Spironolactone*^§^ (6e-3, 2305)  Sulfacetamide*^§^ (4e-3, 2309)  Sulfadiazine* (8e-3, 2301)  Tacrine* (5e-3, 2306)  Terguride^§^ (3e-5, 2354)  Thioproperazine^§^ (7e-3, 2302)  Tolazamide*^§^ (3e-3, 2312)  Tolbutamide*(1e-6, 2376)  Triamterene* (9e-3, 2300)  Triflupromazine^§^ (2e-3, 2317)  Urapidil^§^ (6e-3, 2304) |
| Drugs predicted in both UC/DB and DC/UB | | | |
| Overlap predictions | Benperidol^§^  Chlortetracycline*^§^  Desoxycortone^§^  [Dizocilpine](http://www.springerlink.com/content/4316j55q05086706/)^§^  Domperidone^§^  Fulvestrant (in use)^§^  [Irinotecan*(Ph 2)](http://clinicaltrials.gov/ct2/show/NCT00003351?term=IRINOTECAN&cond=breast+cancer&rank=6)^§^  Nilutamide*^§^  [Sirolimus*(Ph 2)](http://clinicaltrials.gov/ct2/show/NCT00411788?term=SIROLIMUS&cond=breast+cancer&rank=1)^§^  [Valproic acid*(Ph 2)](http://clinicaltrials.gov/ct2/show/NCT01010854?term=VALPROIC+ACID&cond=breast+cancer&rank=2)^§^ | Atropine oxide^§^  Brinzolamide*^§^  Chlorphenamine*^§^  Clenbuterol^§^  Colforsin^§^  Diperodon^§^  [Disulfiram*](http://www.ncbi.nlm.nih.gov/pubmed/17026967)^§^  [Estradiol*](http://www.jbc.org/content/277/47/44772.abstract)^§^  Etoposide^§^ (in use)  Mafenide^§^  Mefloquine*^§^  Mepacrine^§^  Methylergometrine*^§^  Nomifensine^§^  Oxolinic acid^§^  Pyrantel^§^  Thioridazine*^§^  [Tranexamic acid*](http://www3.interscience.wiley.com/journal/122355552/abstract)^§^  Zaprinast^§^ | Adiphenine^§^  Alprenolol^§^  [Alprostadil*(Ph2)](http://clinicaltrials.gov/ct2/show/NCT00080808?term=ALPROSTADIL&cond=prostate+cancer&rank=2) ^§^  Amiprilose^§^  Articaine^§^  Azapropazone^§^  Beclometasone*^§^  Benzathine^§^ benzylpenicillin^§^  Biotin^§^  Brompheniramine*^§^  Cefalotin*^§^  [Chenodeoxycholic acid*](http://www.ncbi.nlm.nih.gov/pubmed/12969788)^§^  Chlormezanone*^§^  Chlortalidone*^§^  Clorsulon^§^  [Danazol*](http://www3.interscience.wiley.com/journal/112723117/abstract)^§^  Dapsone*^§^  Debrisoquine^§^  [Deferoxamine*](http://www.informaworld.com/smpp/content~db=all~content=a790502556)^§^  [Desipramine](http://www.ncbi.nlm.nih.gov/pubmed/18606486)^§^  Diethylstilbestrol^§^(in use)  Dihydroergotamine*^§^  Dioxybenzone^§^  Disopyramide*^§^  [Disulfiram*](http://webcache.googleusercontent.com/search?q=cache:NF7tcE11YBcJ:www.asco.org/ASCOv2/Meetings/Abstracts%3F%26vmview%3Dabst_detail_view%26confID%3D64%26abstractID%3D20452+"DISULFIRAM+"+"prostate+cancer"&cd=1&hl=ja&ct=clnk&gl=jp&client=firefox-a)^§^  Dizocilpine^§^  Domperidone^§^  Ethaverine^§^  Ethionamide*^§^  Flecainide*^§^  [Fluvastatin*](http://mct.aacrjournals.org/content/5/9/2310.long)^§^  Guanabenz*^§^  Guanadrel*^§^  Homochlorcyclizine^§^  [Hydrocortisone*(Ph 3)](http://clinicaltrials.gov/ct2/show/NCT00002881?term=HYDROCORTISONE&cond=prostate+cancer&rank=3)^§^  Iohexol^§^  Isoniazid*^§^  Isoxicam^§^  Levocabastine*^§^  Lidocaine*^§^  Lynestrenol^§^  Mafenide^§^  Mefexamide^§^  Memantine*^§^  Metampicillin^§^  Metergoline^§^  Metixene*^§^  Mianserin^§^  Mometasone*^§^  Moxonidine^§^  [Mycophenolic acid*](http://www.ncbi.nlm.nih.gov/pubmed/16356827)^§^  Naftifine*^§^  Nicergoline^§^  Niclosamide^§^  Nicotinic acid*^§^  [Nifedipine*](http://www.ncbi.nlm.nih.gov/pubmed/15728367)^§^  Ondansetron*^§^  Orphenadrine*^§^  Oxantel^§^  Oxyphenbutazone*^§^  [Paclitaxel* (Ph 3)](http://clinicaltrials.gov/ct2/show/NCT00004054?term=PACLITAXEL&cond=prostate+cancer&rank=19) ^§^  Pergolide*^§^  Perphenazine*^§^  Pimozide*^§^  Propoxycaine^§^  Pyrithyldione^§^  Ribavirin*^§^  [Sirolimus* (Ph 2)](http://clinicaltrials.gov/ct2/show/NCT00922129?term=SIROLIMUS&cond=prostate+cancer&rank=1) ^§^  Sisomicin^§^  Spiperone^§^  Spiramycin^§^  Spironolactone*^§^  Sulfacetamide*^§^  [Sulindac*](http://www.ncbi.nlm.nih.gov/pubmed/9933075)^§^  Tacrine*^§^  [Tanespimycin(Ph 2)](http://clinicaltrials.gov/ct2/show/NCT00118092?term=TANESPIMYCIN&cond=prostate+cancer&rank=2) ^§^  Terguride^§^  Thioproperazine^§^  Tolazamide*^§^  Tolbutamide*^§^  Triflupromazine^§^  Urapidil^§^ |
| Total | 10 | 19 | 82 |
