# Supplementary material for: Using Functional Signatures to Identify Repositioned Drugs for Breast, Myelogenous Leukemia and Prostate Cancer
Source: PLoS Comput Biol. 2012 Feb 9;8(2):e1002347. doi: 10.1371/journal.pcbi.1002347 (PMC3276504; doi:10.1371/journal.pcbi.1002347)
Supplement: Table S2 — KEGG pathways enriched in top up/down regulated genes breast cancer tissue and corresponding down/up regulated genes in response to cell line perturbations with bioactive compounds (see Methods). AD: Adherens junction, B: Bacterial invasion of epithelial cells, D: Drug metabolism - cytochrome P450, E: ErbB signaling pathway, F: Focal adhesion, M: Riboflavin metabolism, N: Nucleotide excision repair, R: Ribosome, T: Thiamine metabolism. (DOC) [file pcbi.1002347.s003.doc]

**Table S2.** KEGG pathways enriched in top up/down regulated genes breast cancer tissue and corresponding down/up regulated genes in response to cell line perturbations with bioactive compounds (see Methods). Color code associates genes with pathways**. AD**: Adherens junction, **B**: Bacterial invasion of epithelial cells, **D**: Drug metabolism - cytochrome P450, **E**: ErbB signaling pathway, **F**: Focal adhesion, **M**: Riboflavin metabolism, **N**: Nucleotide excision repair, **R**: Ribosome , **T**: Thiamine metabolism.

|  | **Drug** | **Genes** | **Pathways** |
| --- | --- | --- | --- |
| UC/DB | ADENOSINE PHOSPHATE | *CAMK2G, CAV1, CAV2, COL5A1, ERCC4, FN1, FYN, LAMB1, RFK, VCL* | **AD, B, E, F, M, N** |
|  | AMILORIDE* | *CAMK2B, CAMK2G, CAV1, COL5A1, ERCC4, FN1, LAMB1, MAPK9, NCK1, PVRL3, RAD23B, VCL* | **AD, B, E, F, N** |
|  | AMOXICILLIN* | *COL5A1, CUL4A, ERCC4, LAMB1, MTMR2, PRKCA, SNAI2* | **AD, E, F, M, N, T** |
|  | BENPERIDOL | *AKT3, ARPC5, , CAV1, CAV2, FN1, IQGAP1, LAMB1, NCK1* | **AD, B, E, F** |
|  | BENSERAZIDE | *CAMK2G, COL5A1, CUL4B, ERCC4, ERCC8, FN1, MTMR1* | **B, E, F, M, N, T** |
|  | CHLORTETRACYCLINE*^§^ | *CAV1, COL5A1, CTNNA1, CUL4B, FN1, NFS1, RFK* | **AD, B, F, M, N, T** |
|  | DESOXYCORTONE | *CAV2, CLTB, COL5A1, CUL4B, EGFR, ERCC4, FN1, LAMB1, MTMR1, NFS1, PRKCA, RAD23B, TJP1* | **AD, B, E, F, M, N, T** |
|  | DEXIBUPROFEN | *COL5A1, ERCC8, FN1, LAMB1, PRKCA, RFK* | **B, E, F, M, N** |
|  | DIZOCILPINE | *ARPC5, CAV2, CUL4B, EGFR, ERCC4, FN1, LAMB1, MTMR2, NFS1, RFK* | **AD, B, E, F, M, N, T** |
|  | DOMPERIDONE | *CAMK2G, CAV1, CAV2, CUL4A, CUL4B, ERCC4, FN1, IQGAP1, MAPK1, NCK1, SNAI2* | **AD, B, E, F, N** |
|  | ESTRADIOL*(Ph 3) | *CAV1, CUL4B, ERCC4, FN1, LAMB1, MAPK1* | **AD, B, E, F, N** |
|  | FULVESTRANT* (in use) | *CAV1, EGFR, LAMC1, NCK1, SNAI2* | **AD, B, E, F** |
|  | GALANTAMINE | *ARPC5, CAV2, CUL4A, EGFR, FN1, IQGAP1, LAMB1, MET* | **AD, B, E, F, N** |
|  | IRINOTECAN*^§^(Ph 2) | *CUL4B, NCK1, PTK2,* TJP1 | **AD, B, E, F, N** |
|  | METERGOLINE | *CAV2, COL5A1, EGFR, ERCC4, ERCC8, LAMB1, MET, NFS1, POLE3, PRKCA, RAD23B* | **AD, B, E, F, N, T** |
|  | NILUTAMIDE*^§^ | *ARPC5, CAV1, CAV2, CUL4B, ERCC4, ERCC8, LAMB1, MAPK9, MTMR2, POLE3, RAD23B, RFK, TJP1* | **AD, B, E, F, M, N, T** |
|  | NOCODAZOLE | *COL5A1, CUL4B, ERCC4, ERCC8, FN1, LAMB1, MAPK9, NCK1, PRKCA* | **B, E, F, N** |
|  | PIRINIXIC ACID | *COL5A1, CRKL, EGFR, FN1, LAMC1, NCK1, PRKCA, PTK2, RAD23B* | **AD, B, E, F, N** |
|  | PROMETHAZINE* | *CAMK2G, COL5A1, ERCC8, MET, MTMR1, NCK1, TJP1* | **AD, B, E, F, M, N, T** |
|  | PROPRANOLOL* | *CAMK2G, CAV1, EGFR, FN1, FYN, MET, NCK1, PVRL3, RAD23B, SNAI2* | **AD, B, E, F, N** |
|  | ROLITETRACYCLINE | *CAMK2G, CAV1, CLTB, ERCC4, FN1, IQGAP1, LAMB1, LAMC1, MTMR2, PRKCA, RFK* | **AD, B, E, F, M, N, T** |
|  | SIROLIMUS*^§^(Ph 3) | *CAMK2G, FN1, MAPK1, NCK1, PRKCA, RAD23B, TJP1* | **AD, B, E, F, N** |
|  | THIORIDAZINE* | *ARPC5, CAMK2G, CAV1, CAV2, ERCC8, MTMR1, NFS1, PRKCA* | **B, E, F, M, N, T** |
|  | TILETAMINE | *CAV2, COL5A1, EGFR, FN1, LAMB1, MET, MTMR2, PVRL3, SNAI2* | **AD, B, E, F, M, T** |
|  | TROLEANDOMYCIN* | *CAV1, CAV2, COL5A1, EGFR, FYN, LAMB1, LAMC1, MTMR2* | **AD, B, E, F, M, T** |
|  | VALPROIC ACID*^§^(Ph 2) | *ARPC5, CAMK2G, CAV1, COL5A1, CUL4B, IQGAP1, MTMR2, NCK1, POLE3* | **AD, B, E, F, M, N, T** |
|  | XYLAZINE | *COL5A1, ERCC4, FN1, IQGAP1, LAMB1, PTK2* | **AD, B, E, F, N** |
|  | ZAPRINAST | *CUL4A, EGFR, ERCC8, FN1, NCK1, PTK2, PVRL3, RFK* | **AD, B, E, F, M, N** |
| DC/UB | ACENOCOUMAROL | *CYP2A6, RPL35A* | **D, R** |
|  | AMIKACIN* | *GSTM2, RPL18, RPL35A, RPS25, RPS28, RPS9* | **D, R** |
|  | ARTEMISININ | *CYP2B6, RPS11, RPS4Y1* | **D, R** |
|  | AZACITIDINE* | *CYP2A6, FMO5, GSTK1, MAOA, RPL18, RPS9* | **D, R** |
|  | BENPERIDOL | *FMO5, RPS4Y1* | **D, R** |
|  | BETAZOLE* | *CYP2A7, FMO5, GSTM2, RPL35A, RPS11, RPS4Y1* | **D, R** |
|  | BUPROPION*(Ph 4) | *RPL35A, RPS11, RPS4Y1* | **R** |
|  | CETIRIZINE* | *CYP2A6, GSTM2, MAOA, RPL14, RPL22, RPL35A, RPS4Y1* | **D, R** |
|  | CHLORPROPAMIDE* | *CYP2A6, FMO5, MAOA, RPL18, RPL35A, RPS11* | **D, R** |
|  | CHLORTETRACYCLINE*^§^ | *CYP2A6,* RPL35A | **D, R** |
|  | CHLORZOXAZONE* | *CYP2A6, CYP2C19, CYP3A7, RPL35A, RPS4Y1* | **D, R** |
|  | CLENBUTEROL | *FMO5, MAOA, RPL35A* | **D, R** |
|  | CLOZAPINE* | *MAOA,* RPL35A | **D, R** |
|  | DEBRISOQUINE | *GSTM2, RPL14, RPS28* | **D, R** |
|  | DESOXYCORTONE | *CYP3A7, RPL35A, RPS18* | **D, R** |
|  | DEXAMETHASONE*  (Ph 3) | *CYP2A7, RPL18, RPL31, RPS9* | **D, R** |
|  | DINOPROSTONE* | *CYP2A6, CYP3A7, RPL18, RPS4Y1, RPS9, UGT2B15* | **D, R** |
|  | DIOXYBENZONE | *CYP2A6, RPL18, RPL35A* | **D, R** |
|  | DIZOCILPINE* | *CYP2A6, RPL18* | **D, R** |
|  | DOMPERIDONE | *CYP2A6, CYP2A7, MAOA,* RPL35A*, RPS11, RPS28, RPS4Y1* | **D, R** |
|  | DYDROGESTERONE* | *FMO5, RPL35A* | **D, R** |
|  | ETOPOSIDE*(Ph 2) | ***CYP2A6****, RPS4Y1* | **D, R** |
|  | ETYNODIOL* | *CYP2A6, MAOA, RPL18, RPS28, UGT2B15* | **D, R** |
|  | EUCATROPINE | *CYP2A7, RPL35A, RPS28, RPS4Y1* | **D, R** |
|  | FELODIPINE* | *RPL35A* | **R** |
|  | FLUOXETINE* | *FMO5, RPS11* | **D, R** |
|  | FULVESTRANT* (in use) | *CYP2C19* | **D** |
|  | GABAPENTIN*(Ph 3) | *GSTM2, UGT2B15* | **D** |
|  | GENTAMICIN* | *FMO5, RPL31, RPL35A, RPS28* | **D, R** |
|  | GUAIFENESIN* | *CYP2A6, CYP3A7, UGT2B15* | **D** |
|  | GUANADREL* | *RPL18, RPL31* | **R** |
|  | HYCANTHONE | *CYP2A7, FMO5, RPL35A* | **D, R** |
|  | IOHEXOL | *RPL35A, RPS4Y1* | **R** |
|  | IRINOTECAN*^§^(Ph 2) | *CYP2A6, GSTK1, RPL15, RPL18, RPL35A, RPS28, RPS9* | **D, R** |
|  | KETANSERIN | *CYP2A6, CYP2A7, RPL22, UGT2B15* | **D, R** |
|  | LORGLUMIDE | *FMO5, RPL35A, RPS4Y1* | **D, R** |
|  | MEFEXAMIDE | *RPL14, RPL18, RPS28* | **R** |
|  | MESTRANOL | *CYP2A6, RPS9* | **D, R** |
|  | METAMPICILLIN | *RPL18, RPL35A* | **R** |
|  | METHOTREXATE*(Ph 3) | *FMO5, GSTK1* | **D** |
|  | MOROXYDINE | *RPL35A, RPS9* | **R** |
|  | MYCOPHENOLIC ACID* | *CYP2A7, GSTM2, RPL18, RPL31, UGT2B15* | **D, R** |
|  | NAPHAZOLINE | *CYP2A6, FMO5, RPL28, RPS28* | **D, R** |
|  | NICARDIPINE* | *CYP2A6, CYP2A7, FMO5* | **D** |
|  | NIFENAZONE | *CYP2A7, FMO5* | **D** |
|  | NIMESULIDE | *RPL18, RPS4Y1, UGT2B15* | **D, R** |
|  | NIMODIPINE* | *CYP2A6, CYP2A7, FMO2, MAOA, RPL18, RPS4Y1, UGT2B15* | **D, R** |
|  | NOMEGESTROL | *RPL18, UGT2B15* | **D, R** |
|  | PHENOXYBENZAMINE* | *CYP3A7, RPL31, RPL35A, RPS28* | **D, R** |
|  | PRIMAQUINE* | *CYP2A7, RPL18, RPL28, RPS4Y1* | **D, R** |
|  | PROCHLORPERAZINE*  (Ph 3) | *CYP2A6, RPL28, RPL31* | **D, R** |
|  | SIROLIMUS*^§^(Ph 2) | *MAOA* | **D** |
|  | TESTOSTERONE*(Ph 2) | *CYP3A7, FMO2, RPL35A* | **D, R** |
|  | TETROQUINONE | *MAOA, RPL35A* | **D, R** |
|  | TOPIRAMATE* | *RPS28, RPS4Y1* | **R** |
|  | TRIFLUOPERAZINE* | *MAOA, RPL31, RPS28, RPS4Y1, RPS9* | **D, R** |
|  | TROGLITAZONE* | *FMO5, RPL31, RPS28, RPS4Y1* | **D, R** |
|  | TUBOCURARINE CHLORIDE* | *FMO5, MAOA, RPL18, RPL35A, RPS28* | **D, R** |
|  | VALPROIC ACID*^§^(Ph 2) | *RPS11, RPS9* | **R** |
| ***** FDA approved drugs**,** ^§^Predictions with duality | | | |
