# Supplementary material for: Using Functional Signatures to Identify Repositioned Drugs for Breast, Myelogenous Leukemia and Prostate Cancer
Source: PLoS Comput Biol. 2012 Feb 9;8(2):e1002347. doi: 10.1371/journal.pcbi.1002347 (PMC3276504; doi:10.1371/journal.pcbi.1002347)
Supplement: Table S3 — KEGG pathways enriched in top up/down regulated genes leukemia and corresponding down/up regulated genes in response to cell line perturbations with bioactive compounds (see Methods). G: Glycerolipid metabolism, GL: Glycerophospholipid metabolism, GPI: Glycosylphosphatidylinositol (GPI)-anchor biosynthesis, VA: Vascular smooth muscle contraction, TGF: TGF-β signaling pathway, C: Cell cycle, A: Apoptosis, TC: T cell receptor signaling. (DOC) [file pcbi.1002347.s004.doc]

**Table S3.** KEGG pathways enriched in top up/down regulated genes leukemia and corresponding down/up regulated genes in response to cell line perturbations with bioactive compounds (see Methods). Color code associates genes with pathways. **G**: Glycerolipid metabolism, **GL**: Glycerophospholipid metabolism, **GPI**: Glycosylphosphatidylinositol (GPI)-anchor biosynthesis, **VA**: Vascular smooth muscle contraction, **TGF**: TGF-βsignaling pathway, **C**: Cell cycle, **A**: Apoptosis, **TC**: T cell receptor signaling.

|  | | | |
| --- | --- | --- | --- |
|  | **Drug** | **Genes involved** | **Pathways** |
| UC/DB | ACEMETACIN | ***DGAT1, PLA2G6*** | **G, GL, VA** |
|  | ACENOCOUMAROL | ***GPAA1, MBOAT2, PLA2G6, TAZ*** | **G, GL, GPI, VA** |
|  | ALFUZOSIN* | ***TGFB3*** | **TGF** |
|  | ALPROSTADIL* | ***PCYT2*** | **GL** |
|  | ALVESPIMYCIN | ***DGAT1, GPAA1, PCYT2, TAZ*** | **G, GL, GPI** |
|  | AMIKACIN* | ***AGPAT1, PLA2G6, TAZ*** | **G, GL, VA** |
|  | ASTEMIZOLE | ***GPAA1, ROCK2*** | **GPI, TGF, VA** |
|  | ATROPINE METHONITRATE | ***ADORA2A, GNA11, PLA2G6, SMURF1*** | **GL, TGF, VA** |
|  | ATROPINE OXIDE^^ | ***ADCY9, GPAA1, MBOAT2, PCYT2, PLA2G6, RAMP3, ROCK2, TFDP1, TGFB3*** | **G, GL, GPI, TGF, VA** |
|  | BENZOCAINE | ***AGPAT1, PCYT2, PIGL*** | **G, GL, GPI** |
|  | BRINZOLAMIDE*^^ | ***AGPAT1, GPAA1, PLA2G6*** | **G, GL, GPI, VA** |
|  | CHLOROQUINE* | ***DCN, PLD1, ROCK2*** | **GL, TGF, VA** |
|  | CHLORPHENAMINE*^^ | ***ACVR2B, ADCY9, GPAA1, RAMP3, ROCK2, TFDP1*** | **GPI, TGF, VA** |
|  | CHLORPROMAZINE* | ***ADORA2A, PRKCE, ROCK2*** | **TGF, VA** |
|  | CIPROFLOXACIN* | ***PIGL, PIGZ, TFDP1*** | **GPI, TGF** |
|  | CLORGILINE | ***ACVR2B, PLA2G6, TGFB3*** | **GL, TGF, VA** |
|  | COLFORSIN^^ | ***ACVR2B, PCYT2, RAMP3*** | **GL, TGF, VA** |
|  | COTININE | ***MBOAT2, PIGL, PIGZ, ROCK2*** | **G, GL, GPI, TGF, VA** |
|  | DEHYDROCHOLIC ACID | ***ADORA2A, MBOAT2, PIGL*** | **G, GL, GPI, VA** |
|  | DESIPRAMINE | ***ACVR2B, ROCK2, TFDP1*** | **TGF, VA** |
|  | DIAZOXIDE* | ***ROCK2*** | **TGF, VA** |
|  | DIHYDROERGOTAMINE* | ***PCYT2*** | **GL** |
|  | DINOPROST | ***ADORA2A, PIGL*** | **GPI, VA** |
|  | DIPERODON^^ | ***GPAA1, MBOAT2, PLA2G6, RAMP3, TGFB3*** | **G, GL, GPI, TGF, VA** |
|  | DISULFIRAM*^^ | ***ACVR2B, ADCY9, GPAA1, MBOAT2, PIGL, ROCK2*** | **G, GL, GPI, TGF, VA** |
|  | DOSULEPIN | ***DCN*** | **TGF** |
|  | DOXYLAMINE | ***TGFB3*** | **TGF** |
|  | ENOXACIN* | ***ACVR2B, GPAA1*** | **GPI, TGF** |
|  | ESTRADIOL*^^ | ***ACVR2B, ADCY9, AGPAT1, DCN, MBOAT2, RAMP3, TAZ*** | **G, GL, TGF, VA** |
|  | ETODOLAC* | ***ROCK2*** | **TGF, VA** |
|  | ETOPOSIDE*(in use) | ***PCYT2*** | **GL** |
|  | FUROSEMIDE* | ***DCN, GPAA1, RAMP3, TAZ*** | **GL, GPI, TGF, VA** |
|  | GLIPIZIDE* | ***MBOAT2, PIGZ*** | **G, GL, GPI** |
|  | HALOPERIDOL* | ***DCN, GNA11, PIGZ, ROCK2*** | **GPI, TGF, VA** |
|  | HYCANTHONE | ***ROCK2, TFDP1*** | **TGF, VA** |
|  | ISOCONAZOLE | ***ROCK2*** | **TGF, VA** |
|  | ISONIAZID* | ***DGAT1, PCYT2, PIGL, PLA2G6, RAMP3, ROCK2*** | **G, GL, GPI, TGF, VA** |
|  | IVERMECTIN* | ***ACVR2B, PLA2G6*** | **GL, TGF, VA** |
|  | LOXAPINE* | ***ADORA2A, DCN, MBOAT2, PIGL, ROCK2*** | **G, GL, GPI, TGF, VA** |
|  | MAFENIDE^^ | ***ACVR2B, DCN, DGAT1, PLA2G6, TAZ*** | **G, GL, TGF, VA** |
|  | MEFLOQUINE*^^ | ***TFDP1*** | **TGF** |
|  | MEPACRINE | ***LPL, PCYT2*** | **G, GL** |
|  | MEPENZOLATE BROMIDE* | ***ADCY9, PLA2G6, PRKCE*** | **GL, VA** |
|  | METERGOLINE | ***ACVR2B, PCYT2*** | **GL, TGF** |
|  | METITEPINE | ***ADORA2A, GPAA1, PLA2G6*** | **GL, GPI, VA** |
|  | METRIZAMIDE* | ***ACVR2B, GPAA1*** | **GPI, TGF** |
|  | MICONAZOLE* | ***TAZ, TFDP1, TGFB3*** | **GL, TGF** |
|  | MINOCYCLINE* | ***ADCY9, PIGL*** | **GPI, VA** |
|  | MINOXIDIL* | ***PCYT2, PLA2G6, TFDP1*** | **GL, TGF, VA** |
|  | MOLSIDOMINE | ***ADORA2A, TFDP1*** | **TGF, VA** |
|  | MOMETASONE* | ***ADORA2A, DCN, PIGL*** | **GPI, TGF, VA** |
|  | NABUMETONE* | ***ADCY9, ROCK2, SMURF1*** | **TGF, VA** |
|  | NALTREXONE* | ***PIGL, ROCK2, TFDP1*** | **GPI, TGF, VA** |
|  | NICARDIPINE* | ***GPAA1, PLA2G6, ROCK2, TGFB3*** | **GL, GPI, TGF, VA** |
|  | NOMIFENSINE^^ | ***AGPAT1, MBOAT2, ROCK2*** | **G, GL, TGF, VA** |
|  | NORFLOXACIN | ***ADCY9, ROCK2*** | **TGF, VA** |
|  | ORCIPRENALINE* | ***ACVR2B, MBOAT2*** | **G, GL, TGF** |
|  | OXOLINIC ACID^^ | ***PCYT2*** | **GL** |
|  | OXYBUPROCAINE* | ***DGAT1, TAZ*** | **G, GL** |
|  | OXYBUTYNIN | ***ADORA2A, MBOAT2, PIGL, RAMP3, TAZ, TFDP1*** | **G, GL, GPI, TGF, VA** |
|  | PENTETRAZOL | ***PCYT2*** | **GL** |
|  | PERGOLIDE* | ***PCYT2*** | **GL** |
|  | PERPHENAZINE* | ***GPAA1, ROCK2*** | **GPI, TGF, VA** |
|  | PHENINDIONE* | ***DCN, PLD1*** | **GL, TGF** |
|  | PINDOLOL* | ***PCYT2, PIGL, PLA2G6*** | **GL, GPI, VA** |
|  | PREDNISONE* (in use) | ***ACVR2B, ADORA2A, DGAT1, PLA2G6, TAZ, TFDP1, TGFB3*** | **G, GL, TGF, VA** |
|  | PUROMYCIN | ***ACVR2B, PLA2G6*** | **GL, TGF, VA** |
|  | PYRIDOXINE* | ***DCN, MBOAT2, PCYT2*** | **G, GL, TGF** |
|  | PYRITHYLDIONE | ***ADCY9*** | **VA** |
|  | STREPTOMYCIN* | ***ACVR2B, MBOAT2, TGFB3*** | **G, GL, TGF** |
|  | SULFADIAZINE* | ***PCYT2, PLD1, ROCK2*** | **GL, TGF, VA** |
|  | SULPIRIDE | ***TGFB3*** | **TGF** |
|  | TAMOXIFEN* | ***ACVR2B, MBOAT2, ROCK2, TFDP1, TGFB3*** | **G, GL, TGF, VA** |
|  | TANESPIMYCIN | ***TAZ*** | **GL** |
|  | THALIDOMIDE* | ***ACVR2B, ADCY9, DCN, ROCK2*** | **TGF, VA** |
|  | THIOPROPERAZINE* | ***MBOAT2, ROCK2, TFDP1*** | **G, GL, TGF, VA** |
|  | THIORIDAZINE*^^ | ***MBOAT2, PCYT2*** | **G, GL** |
|  | TICLOPIDINE* | ***ACVR2B, GPAA1, TFDP1*** | **GPI, TGF** |
|  | TRANEXAMIC ACID*^^ | ***ADORA2A, RAMP3, ROCK2*** | **TGF, VA** |
|  | TRIFLUSAL | ***ADORA2A, PIGL, ROCK2*** | **GPI, TGF, VA** |
|  | YOHIMBIC ACID | ***ADCY9, PLA2G6*** | **GL, VA** |
|  | ZAPRINAST^^ | ***ROCK2*** | **TGF, VA** |
| DC/UB | ATROPINE OXIDE^^ | ***BUB3, CDKN1B,VAV3*** | **C, TC** |
|  | BRINZOLAMIDE* | ***BUB3, CDC27, CDK7*** | **C** |
|  | BROMOCRIPTINE* | ***CD8A, CDKN2C, CFLAR, PTPRC, TNFSF10*** | **A, C, TC** |
|  | CHLORPHENAMINE*^^ | ***CDKN1B*** | **C** |
|  | CLENBUTEROL | ***ATM, CAPN2, CFLAR, PTPRC*** | **A, C, TC** |
|  | COLFORSIN | ***CDKN1B, CDKN2C, CFLAR, PTPRC*** | **A, C, TC** |
|  | DIFLUNISAL* | ***CDC42, CDK7, CFLAR*** | **A, C, TC** |
|  | DIPERODON | ***CFLAR, PTPRC*** | **A, TC** |
|  | DISULFIRAM* | ***CDC27, CDKN2C, FAS, TNFRSF10B*** | **A, C** |
|  | ESTRADIOL*^^ | ***BUB3, CAPN2, CDKN1B, CDKN2C*** | **A, C** |
|  | ETOPOSIDE* (in use) | ***BIRC3, CFLAR, PTPRC, TNFSF10,*** | **A, TC** |
|  | LANATOSIDE C | ***BIRC3, CFLAR*** | **A** |
|  | MAFENIDE^^ | ***CFLAR, PDK1*** | **A, TC** |
|  | MEFLOQUINE*^^ | ***CDKN1B, CFLAR, KRAS*** | **A, C, TC** |
|  | MEPACRINE | ***CDC42, CDKN1B, KRAS*** | **C, T** |
|  | METHYLERGOMETRINE* | ***CDKN2C, CFLAR, PTPRC, TNFSF10*** | **A, C, TC** |
|  | NEOMYCIN* | ***BUB3, CASP8, CFLAR, SMC3*** | **A, C** |
|  | NICLOSAMIDE | ***BIRC3, CDKN1B, CFLAR, TNFRSF10B*** | **A, C** |
|  | NOCODAZOLE | ***CAPN2, CDC42, CFLAR, KRAS, PTPRC, TNFRSF10B*** | **A, TC** |
|  | NOMIFENSINE | ***ANAPC5, CD3D*** | **C, TC** |
|  | OXOLINIC ACID | ***BCL2, BIRC2, CASP8, DLG1, FAS , PDK1*** | **A, TC** |
|  | PYRANTEL | ***DLG1, PDK1, TNFRSF10B*** | **A, TC** |
|  | SULOCTIDIL | ***BIRC3, CFLAR, ENDOD1, FAS, SMAD3, TNFSF10*** | **A, C** |
|  | THIORIDAZINE* | ***ATM, BUB3, CDK7, FAS, FYN, KRAS, TNFRSF10B, , VAV3*** | **A, C, TC** |
|  | TRANEXAMIC ACID* | ***BUB3, CDKN1B*** | **C** |
|  | ZAPRINAST | ***CDKN1B, PTPRC*** | **C, TC** |
| ***** FDA approved drugs**,** ^^Predictions with duality | | | |
