# Supplementary material for: Using Functional Signatures to Identify Repositioned Drugs for Breast, Myelogenous Leukemia and Prostate Cancer
Source: PLoS Comput Biol. 2012 Feb 9;8(2):e1002347. doi: 10.1371/journal.pcbi.1002347 (PMC3276504; doi:10.1371/journal.pcbi.1002347)
Supplement: Table S4 — GO terms enriched in top up/down regulated genes in breast cancer tissue for the window size specified in Table 1. (DOC) [file pcbi.1002347.s005.doc]

**Table S4.** GO terms enriched in top up/down regulated genes in breast cancer tissue for the window size specified in Table 1.

|  |  | Enriched GO terms | Total |
| --- | --- | --- | --- |
| Shared term between UC/DB and DC/UB | Biological process | Anatomical structure formation involved in morphogenesis  Carboxylic acid metabolic process  Cell adhesion  Cell development  Cell-cell signaling  Cellular component morphogenesis  Cellular ion homeostasis  Cellular macromolecular complex subunit organization  Cellular membrane organization  Cellular response to chemical stimulus  DNA metabolic process  Immune response  Multi-organism process  Nucleobase, nucleoside, nucleotide and nucleic acid metabolic process  Organ morphogenesis  Protein complex assembly  Regulation of anatomical structure size  Regulation of cell cycle  Regulation of cell differentiation  Regulation of cellular component organization  Regulation of cellular protein metabolic process  Regulation of multicellular organismal development  Regulation of signaling pathway  Regulation of transcription from RNA polymerase II promoter  Regulation of transport  Response to abiotic stimulus  Response to biotic stimulus  Response to DNA damage stimulus  Response to hormone stimulus  Response to wounding  Small molecule biosynthetic process  Small molecule catabolic process  Transmembrane transport | 33 |
|  | Cellular component | Cell fraction  Cell projection  Cytoplasmic membrane-bounded vesicle  Cytoskeletal part  Cytosol  Endoplasmic reticulum membrane  Extracellular space  Integral to plasma membrane  Membrane  Membrane fraction  Protein complex | 11 |
|  | Molecular function | ATP binding  GTPase regulator activity  Identical protein binding  Protein binding  Protein dimerization activity  Protein serine/threonine kinase activity  Structural molecule activity  Transcription factor activity  Zinc ion binding | 9 |
| UC/DB | Biological process | Amine metabolic process (**T**)  Cell migration (**AD, E**)  Cellular macromolecule catabolic process  Cellular nitrogen compound biosynthetic process  Cellular nitrogen compound metabolic process  Intracellular protein transport  Ligase activity  M phase  Mitotic cell cycle  Negative regulation of apoptosis  Oxidation reduction  Protein catabolic process  Purine nucleotide metabolic process  Regulation of response to stimulus  Translation  Vesicle-mediated transport | 16 |
|  | Cellular component | Chromosomal part  Golgi apparatus  Golgi membrane  Nuclear lumen  Nucleolus  Nucleoplasm  Nucleoplasm part | 7 |
|  | Molecular function | Cation transmembrane transporter activity  Hydrolase activity, acting on ester bonds  Lipid binding  Nucleoside-triphosphatase activity  Oxidoreductase activity  RNA binding | 6 |
| DC/UB | Biological process | Cation transport  Cell communication  Cell projection organization  Central nervous system development  Defense response (**D**)  Embryo development  Gamete generation  Growth  Metal ion transport  Negative regulation of transcription (**R**)  Negative regulation of transcription, DNA-dependent (**R**)  Positive regulation of transcription, DNA-dependent (**R**)  Regulation of catalytic activity  Regulation of phosphorylation  Regulation of signal transduction (**R**)  Regulation of transferase activity (**R**)  Tissue development  Transmission of nerve impulse | 18 |
|  | Cellular component | Nucleus | 1 |
|  | Molecular function | Peptidase activity, acting on l-amino acid peptides  Sequence-specific DNA binding  Transcription activator activity (**R**)  Transcription factor binding | 4 |
