# Supplementary material for: Using Functional Signatures to Identify Repositioned Drugs for Breast, Myelogenous Leukemia and Prostate Cancer
Source: PLoS Comput Biol. 2012 Feb 9;8(2):e1002347. doi: 10.1371/journal.pcbi.1002347 (PMC3276504; doi:10.1371/journal.pcbi.1002347)
Supplement: Table S5 — GO terms enriched in top up/down regulated genes in leukemic tissue for the window size specified in Table 1. (DOC) [file pcbi.1002347.s006.doc]

**Table S5**. GO terms enriched in top up/down regulated genes in leukemic tissue for the window size specified in Table 1.

|  |  | Enriched GO terms | Total |
| --- | --- | --- | --- |
| Shared term between UC/DB and DC/UB | Biological process | Cellular protein localization  Multi-organism process  Protein transport  Regulation of cellular component organization  Regulation of phosphorylation  Regulation of signal transduction  Regulation of signaling pathway  Regulation of transport  Response to organic substance | 9 |
|  | Cellular component | Membrane fraction  Nuclear lumen  Nucleoplasm part  Protein complex | 4 |
| UC/DB | Biological process | Cell-cell signaling (**E**)  Positive regulation of catalytic activity  Regulation of response to stimulus (**TGF**)  Transmembrane transport (**E**) | 4 |
|  | Cellular component | Cell junction  Integral to plasma membrane (**E**) | 2 |
|  | Molecular function | ATP binding  Cation transmembrane transporter activity  Identical protein binding  Protein kinase activity (**TGF**) | 4 |
| DC/UB | Biological process | Catabolic process  Cellular catabolic process  Cellular response to stress  Intracellular transport (**C**)  Macromolecule catabolic process  Negative regulation of transcription (**C**)  Positive regulation of transcription, dna-dependent  Regulation of apoptosis (**A**)  Regulation of cell differentiation (**A, T**) | 9 |
|  | Cellular component | Cell fraction  Cytosol | 2 |
|  | Molecular function | Nucleoside-triphosphatase activity  Zinc ion binding | 2 |
