# Supplementary material for: Using Functional Signatures to Identify Repositioned Drugs for Breast, Myelogenous Leukemia and Prostate Cancer
Source: PLoS Comput Biol. 2012 Feb 9;8(2):e1002347. doi: 10.1371/journal.pcbi.1002347 (PMC3276504; doi:10.1371/journal.pcbi.1002347)
Supplement: Table S6 — Enriched KEGG pathways for breast cancer and leukemia and the corresponding p-value. (DOC) [file pcbi.1002347.s007.doc]

**Table S6**. Enriched KEGG pathways for breast cancer and leukemia and the corresponding p-value.

| Cancer type | Correlation | Over-represented pathway | p-value |
| --- | --- | --- | --- |
| Breast cancer | UC/DB | **AD**: Adherens junction | 9e-4 |
|  |  | **B**: Bacterial invasion of epithelial cells | 7e-4 |
|  |  | **E**: ErbB signaling pathway | 3e-3 |
|  |  | **F**: Focal adhesion | 8e-3 |
|  |  | **M**: Riboflavin metabolism | 9e-3 |
|  |  | **N**: Nucleotide excision repair | 8e-3 |
|  |  | **R**: Ribosome | 7e-5 |
|  | DC/UB | **T**: Thiamine metabolism | 2e-3 |
|  |  | **D**: Drug metabolism - cytochrome P450 | 1e-3 |
| Leukemia | UC/DB | **G**: Glycerolipid metabolism | 2e-2 |
|  |  | **GL**: Glycerophospholipid metabolism | 8e-3 |
|  |  | **GPI**: Glycosylphosphatidylinositol (GPI)-anchor biosynthesis | 2e-2 |
|  |  | **VA**: Vascular smooth muscle contraction, | 1e-2 |
|  |  | **TGF**: TGF-βsignaling pathway, | 1e-2 |
|  | DC/UB | **C**: Cell cycle | 3e-3 |
|  |  | **A**: Apoptosis | 7e-6 |
|  |  | **TC**: T cell receptor signaling | 1e-3 |
