# Supplementary material for: Using Functional Signatures to Identify Repositioned Drugs for Breast, Myelogenous Leukemia and Prostate Cancer
Source: PLoS Comput Biol. 2012 Feb 9;8(2):e1002347. doi: 10.1371/journal.pcbi.1002347 (PMC3276504; doi:10.1371/journal.pcbi.1002347)
Supplement: Table S7 — Sensitivity and the specificity for optimal values of window size, k. (DOC) [file pcbi.1002347.s008.doc]

**Table S7.** Sensitivity and the specificity for optimal values of window size, k*

|  |  | With filtering | | | Without filtering | | |
| --- | --- | --- | --- | --- | --- | --- | --- |
| Cancer type | Genes compared | Optimal k | Specificity | Sensitivity | Optimal k | Specificity | Sensitivity |
| Breast cancer | UC /DB | 1200 | 0.97 | 0.25 | 2200 | 0.94 | 0.25 |
|  | DC / UB | 1400 | 0.93 | 0.25 | 900 | 0.94 | 0.25 |
| Leukemia | UC / DB | 700 | 0.90 | 0.40 | 500 | 0.98 | 0.20 |
|  | DC / UB | 800 | 0.97 | 0.20 | 800 | 0.97 | 0.20 |
| Prostate cancer | DC / UB | 5200 | 0.90 | 0.33 | 4200 | 0.69 | 0.33 |
|  | UC /DB | 7000 | 0.90 | 0.33 | 3600 | 0.65 | 0.33 |

*Notation: UC (DC) k highest-ranking (lowest ranking) genes in cancer tissue; UB (DB) k highest ranking (lowest-ranking) genes is treated cell lines.
